# Temporal Contrast Sensitivity Increases despite Photoreceptor Degeneration in a Mouse Model of Retinitis Pigmentosa

**DOI:** 10.1523/ENEURO.0020-21.2021

**Published:** 2021-04-13

**Authors:** Rose L. Pasquale, Ying Guo, Yumiko Umino, Barry Knox, Eduardo Solessio

**Affiliations:** Department of Ophthalmology and Visual Sciences, Center for Vision Research, State University of New York Upstate Medical University, Syracuse, NY 13210

**Keywords:** mouse model, P23H, retinitis pigmentosa, rhodopsin, rod photoreceptors, temporal contrast sensitivity

## Abstract

The detection of temporal variations in amplitude of light intensity, or temporal contrast sensitivity (TCS), depends on the kinetics of rod photoresponse recovery. Uncharacteristically fast rod recovery kinetics are facets of both human patients and transgenic animal models with a P23H rhodopsin mutation, a prevalent cause of retinitis pigmentosa (RP). Here, we show that mice with this mutation (Rho^P23H/+^) exhibit an age-dependent and illumination-dependent enhancement in TCS compared with controls. At retinal illumination levels producing ≥1000 R*/rod/s or more, postnatal day 30 (P30) Rho^P23H/+^ mice exhibit a 1.2-fold to 2-fold increase in retinal and optomotor TCS relative to controls in response to flicker frequencies of 3, 6, and 12 Hz despite significant photoreceptor degeneration and loss of flash electroretinogram (ERG) b-wave amplitude. Surprisingly, the TCS of Rho^P23H/+^ mice further increases as degeneration advances. Enhanced TCS is also observed in a second model (rhodopsin heterozygous mice, Rho^+/−^) with fast rod recovery kinetics and no apparent retinal degeneration. In both mouse models, enhanced TCS is explained quantitatively by a comprehensive model that includes photoresponse recovery kinetics, density and collecting area of degenerating rods. Measurement of TCS may be a non-invasive early diagnostic tool indicative of rod dysfunction in some forms of retinal degenerative disease.

## Significance Statement

Retinal degeneration in humans causes loss of retinal cells, loss of retinal function, and eventual blindness. Understanding the retinal and visual changes that occur early in retinal degenerative disease is critical for improving therapeutic strategies and treatment outcomes. We show here an enhanced ability to detect flickering lights that develops during early retinal degeneration in a mouse model of a human disease. This surprising gain-of-function was caused by a pathologic acceleration of the temporal properties of rod photoresponses. In humans, advanced rod dysfunction is currently diagnosed using full-field electroretinogram (ERG) and perimetry. Measurement of retinal or visual sensitivity to flickering lights such as used here could prove useful as a rapid test for early rod dysfunction in retinal degeneration.

## Introduction

Retinitis pigmentosa (RP) is an inherited retinal degenerative disease affecting ∼1:4000 people worldwide (Pagon, 1988; https://www.nei.nih.gov/). RP is characterized by progressive rod and cone photoreceptor degeneration resulting in gradual vision loss and eventual blindness. Clinical features and progression of RP are variable because of the large degree of genetic heterogeneity underlying the disease (for review, see Hartong et al., 2006; Verbakel et al., 2018). A major cause of RP arises from mutations in rhodopsin (Athanasiou et al., 2018), the light sensitive protein in rod photoreceptors. The most prevalent is a proline to histidine substitution at position 23 (P23H), accounting for ∼15% of autosomal dominant RP (adRP) cases in North America (Dryja et al., 1990; Sung et al., 1991).

The heterozygous P23H rhodopsin knock-in mouse (Rho^P23H/+^; Sakami et al., 2011) recapitulates key features of the adRP phenotype: slow, progressive loss of rod photoreceptors, and scotopic flash electroretinogram (ERG) function followed by secondary loss of cone photoreceptors, and photopic flash ERG function (Sakami et al., 2011; Chiang et al., 2015). Even before major rod degeneration is apparent, the photoresponses of Rho^P23H/+^ rods exhibit uncharacteristically fast recovery kinetics (Sakami et al., 2014). Such accelerated rod recovery kinetics have also been reported in human patients with adRP (Wen et al., 2011, 2012), and in other animal models with disease causing rhodopsin mutations: Rho^P347L/S/+^ pigs (Kraft et al., 2005), Rho^G90D/+^ mice (Sieving et al., 2001; Woodruff et al., 2007), Rho^E150K^ mice (Zhang et al., 2013), and reduced rhodopsin expression: Rho^+/−^ mice (Lem et al., 1999; Calvert et al., 2001). However, the impact that the acceleration of rod recovery kinetics has on visual performance during retinal degeneration has not been examined.

The ability to detect temporal variations in light, temporal contrast sensitivity (TCS), has been studied in mice at both the retinal and behavioral levels and is strongly dependent on the response kinetics of rod photoreceptors (Umino et al., 2019). Deceleration of rod photoresponse kinetics by genetic elimination of RGS9 (Chen et al., 2000; Lyubarsky et al., 2001; Keresztes et al., 2004) leads to a profound loss in both retinal and behavioral TCS of mice (Umino et al., 2012). Conversely, acceleration of the rod photoresponse kinetics by genetic overexpression of RGS9 (Krispel et al., 2006; Burns and Pugh, 2009; Chen et al., 2010, 2012) leads to a significant increase in retinal and behavioral TCS (Umino et al., 2019). The increase in TCS occurs only under mesopic light levels, where both rods and cones are active, but is absent at dim (scotopic) light levels (Umino et al., 2012, 2019; Peinado Allina et al., 2017). At mesopic light levels rods operate near their saturation limit with compressed response amplitudes (Umino et al., 2019). Acceleration of their response kinetics leads to shorter integration times (Krispel et al., 2006) and loss of sensitivity to steady background illumination (Fortenbach et al., 2015). This causes an increase in their response bandwidth, a shift in their compressive response attenuation, and thus the increase in TCS (Umino et al., 2019). Whether the link between photoresponse kinetics and TCS extends to mice with disease-causing rhodopsin mutations that speed up rod photoresponse kinetics is unknown.

Here, we investigated retinal and optomotor TCS in Rho^P23H/+^ mice, a knock-in model that exhibits progressive degeneration and accelerated rod recovery kinetics (Sakami et al., 2011, 2014). We used heterozygous (Rho^P23H/+^) rather than homozygous (Rho^P23H/P23H^) mice because (1) most human patients are heterozygous for this mutation and (2) Rho^P23H/P23H^ mice exhibit very rapid retinal degeneration, unlike the Rho^P23H/+^ mice or humans with this form of RP (Sakami et al., 2011). To distinguish effects on TCS arising from degeneration or faster photoresponse kinetics, we also compared TCS in Rho^P23H/+^ mice with that of hemizygous Rho^+/−^ mice which exhibit accelerated rod recovery kinetics but minimal retinal degeneration (Lem et al., 1999; Calvert et al., 2001). We measured retinal TCS using *in vivo* flicker ERGs (Krishna et al., 2002; Shirato et al., 2008; Umino et al., 2019), behavioral TCS using an optomotor assay (Prusky et al., 2004; Umino et al., 2008, 2012) and rod photoresponse kinetics using transretinal ERGs (Vinberg and Koskelainen, 2010). Despite ongoing degeneration, retinal and behavioral TCS of Rho^P23H/+^ mice were significantly enhanced compared with controls as late as postnatal day (P)90. In addition, Rho^+/−^ mice exhibited enhanced TCS before significant retinal degeneration. In both mouse models, enhanced TCS is explained quantitatively by a comprehensive model that includes photoresponse recovery kinetics, density and collecting area of degenerating rods. Thus, altered photoresponse kinetics led to an increase in TCS at mesopic light levels, establishing this paradigm for genetically modified mice, both benign and pathologic. Together, our results suggest that measurement of TCS may be a useful, non-invasive diagnostic tool for diseases that lead to acceleration of rod photoresponse recovery kinetics.

## Materials and Methods

### Animals

The following strains of mice (male and female, one to four months old) were maintained on a C57BL/6J background (The Jackson Laboratory): P23H rhodopsin knock-in (Rho^P23H/+^; Sakami et al., 2011), rhodopsin hemizygote (Rho^+/−^; Lem et al., 1999), cone photoreceptor function loss 3 (GNAT2^cpfl3/cfpl3^; Chang et al., 2006), and double mutant Rho^P23H/+^::GNAT2^cpfl3/cpfl3^ (crossed in our lab). Littermate age-matched Rho^+/+^ and Rho^+/+^::GNAT2^cpfl3/cpfl3^ mice were used as controls. Mice were housed on a 14/10 h light/dark cycle and were provided food and water *ad libitum*. All experiments were performed during the day between zeitgeber times 2 and 10. All procedures were in compliance with both the Guide for the Care and Use of Laboratory Animals and the Association for Research in Vision and Ophthalmology Statement for the Use of Animals in Ophthalmic and Vision Research and were approved by the Institutional Animal Care and Use Committee at SUNY Upstate Medical University.

### Spectral domain optical coherence tomography (SD-OCT) imaging

Three-dimensional images of the retina were acquired using SD-OCT (Envisu-R system: Bioptigen/Leica Microsystems) fitted with a mouse retina probe (50° field of view and 2.5-μm lateral resolution). Before the procedure, mice were anesthetized by intraperitoneal injection of a ketamine/xylazine mixture (90 and 9 mg/kg, respectively). Pupils were dilated with 1% tropicamide and corneas were kept moist with GenTeal lubricant eye gel. Acquired mages were centered on the optic nerve, covering a retinal area of 1.4 × 1.4 mm^2^. Radial volume scans (1000 A-scans/B-scan, 4 B-scans/volume, 40 frames/B-scan) were performed to obtain high resolution images along both the nasal-temporal and dorsal-ventral axes of the retina. For each image, frames were averaged, and horizontal and vertical scale bars were set using calipers in Bioptigen Porter Reader 2.2 software. ImageJ software was used to measure the outer nuclear layer (ONL) thickness at 100-μm intervals starting at 200 μm away from the center of the optic nerve head.

### *In vivo* ERGs

*In vivo* ERGs were measured using a Ganzfeld ColorDome stimulator and Espion E^2^ system (Diagnosys). Mice were dark-adapted overnight, and all procedures were performed under dim red or infrared illumination. Mice were anesthetized with a ketamine/xylazine mixture (90 and 9 mg/kg, respectively) and pupils were dilated with 1% tropicamide. Mouse body temperature was held at 37°C using a heating pad. A reference electrode was placed in the mouth and an intradermal ground electrode was placed next to the tail. A drop of 2.5% hypromellose GONAK solution (AKORN) was applied to the eye. Recordings were performed with gold loop electrodes placed in contact with the cornea. Following setup, mice were dark-adapted an additional 10 min prior the start of the recordings.

Scotopic (dark-adapted) flash ERG stimuli consisted of brief (4 ms in duration) green (∼530 nm) flashes of variable intensity ranging from −5 to 2 log cd*s/m^2^. To minimize the effects of light adaptation, flashes were presented from dimmest to brightest and the time between flashes increased with intensity. The a-wave (measured from the baseline to the a-wave trough) and b-wave (measured from the a-wave trough to the b-wave peak) response amplitudes were analyzed offline using Diagnosys software tools and plotted with SigmaPlot (Systat, Software Inc). Flicker ERGs were evoked by a green (∼530 nm) sinusoidal stimulus at varying levels of mean luminance (−2 to 2 log cd/m^2^), temporal frequency (1.5, 3, 6, 12, and 24 Hz), and contrast (100%, 75%, 50%, 25%, and 0%). For each background intensity, the sequence began with a 3-min light-adaption period. This was followed by flicker stimulation at 100%, 75%, 50%, 25%, and 0% contrast at each temporal frequency. Each flicker trial was ∼4 s in duration for 1.5-Hz flicker stimulation and ∼2 s in duration for 3-, 6-, 12-, and 24-Hz flicker stimulation. The response is an average of 30 trials at each condition. Conversion from luminance to rate of rhodopsin excitation was performed by assuming that 1 (scot) cd/m^2^ generates 800 R*/rod/s and the pupil area was 4 mm^2^ (Lyubarsky et al., 2004; Umino et al., 2019). Retinal intensity levels are expressed in terms of the expected photoisomerization rate (R*/rod/s) for WT rods. A Fourier transform analysis was performed to determine the fundamental magnitude (F0) of the response at each condition. Flicker ERG data were analyzed offline and plotted using SigmaPlot software.

### Determination of retinal TCS

To determine retinal TCS, we plotted the fundamental magnitude as a function of the percent contrast of the sinusoidal stimulus on a log-log axis. Plots of fundamental magnitude versus flicker contrast of P30 Rho^+/+^ control and Rho^P23H/+^ mice were linear on a log-log axis and average slope values ranged between 1.35 and 1.75, commensurate with a mild expansion of the responses to high contrasts. Using these plots, we determined TCS as the inverse of the contrast necessary to reach a threshold level of 10 μVs. The contrast required to elicit this threshold magnitude is considered the threshold contrast. Retinal TCS is the inverse of this threshold contrast and was determined for each individual mouse at each temporal frequency. Retinal TCS was then plotted as a function of temporal frequency to obtain retinal TCS functions (TCSFs). All retinal TCS measurements were performed at a mean background illumination of 2 cd/m^2^ (∼1250 R*/rod/s for control mice).

### Determination of optomotor TCS

Visual acuity and optomotor contrast sensitivity of mice were determined by measuring their optomotor responses to a rotating sine wave grating stimulus using the OptoMotory system (Prusky et al., 2004). The optomotor response is a reflexive head movement of mice tracking the direction of movement of the rotating stimulus. Mice were placed on a pedestal at the center of a testing chamber formed by four computer monitors which displayed the stimulus: vertically oriented, sinusoidal patterned gratings rotating in a clockwise or counter-clockwise direction The observer was blinded to the genotype of the animal and the direction of the stimulus rotation and selected the direction of rotation based on the head movements of the mice, receiving auditory feedback indicating whether the selected direction was correct or incorrect. Trial durations were 5 s. A computer program controlled contrast of the stimulus following a staircase paradigm (Umino et al., 2006) that converged to a threshold value arbitrarily defined as 70% correct responses (Prusky et al., 2004).

Contrast sensitivity was defined as the reciprocal of the threshold contrast value. Contrast sensitivity was measured at temporal frequencies ranging from 0.4 to 12 Hz by varying the speed of rotation (0.5–48°/s) at a constant spatial frequency of 0.236 cycles/°. Sensitivity of each mouse and each condition were the average of four independent trials. All measurements were performed at the unattenuated maximal luminance of the OptoMotry system (∼70 cd/m^2^ or equivalently, ∼1500 R*/rod/s for control mice).

### *Ex vivo* transretinal ERGs

*Ex vivo* ERGs (Vinberg et al., 2014) were performed using a commercially available ERG adapter/specimen holder (Xenotec Inc, Occuscience) connected to the Espion E^2^ system and ColorDome Ganzfeld stimulator (Diagnosys). *Ex vivo* ERGs were performed as described previously (Vinberg et al., 2014; Vinberg and Kefalov, 2015). Briefly, the specimen holder was prepared by filling the electrode channels with electrode solution (140 mm NaCl, 3.6 mm KCl, 2.4 mm MgCl_2_, 1.2 mm CaCl_2_, 3 mm HEPES, and 0.01 mm EDTA, adjusted to a pH 7.4–7.5 using NaOH). Mice were euthanized by cervical dislocation following an intraperitoneal injection of a ketamine/xylazine solution (see above). Eyes were enucleated and retinas were dissected in fresh retina perfusion solution consisting of bicarbonate-containing Ames’ solution [Ames’ media (Sigma-Aldrich, A1420) and 1.9 g of NaHCO_3_ (Sigma-Aldrich, S8875)] bubbled with 95% O_2_/5% CO_2_ and warmed to 37°C. Dissected retinas with retinal pigment epithelium removed were mounted photoreceptor side up on the domes of the specimen holder. The specimen holder was placed inside the ColorDome Ganzfeld stimulator, the recording electrodes were connected, and the retinas were perfused with retina perfusion solution bubbled with 95% O_2_/5% CO_2_ and warmed to 37°C at a flow rate of ∼1.2 ml/min. To isolate photoreceptor responses, a mixture of blockers was added to the perfusion solution. The mixture contained 50 μm of DL-AP4, 20 μm of CNQX, and 100 μm of BaCl_2_ per 100 ml of retina perfusion solution, to block postsynaptic ON bipolar cell, OFF bipolar cell, and glial responses, respectively (Vinberg et al., 2014; Vinberg and Kefalov, 2015). Recordings were started ∼30 min after blockers were added.

The dominant time constant (τ_D_) of the *ex vivo* ERG was determined as described previously (Vinberg and Koskelainen, 2010). A series of flashes of increasing intensity were presented in darkness. We measured the time it took for saturated photoresponses to recover to a threshold value of ∼60% (T_sat_). T_sat_ was then plotted as a function of the natural logarithm of the flash intensity and the slope of this relationship reflects the τ_D_ of the *ex vivo* ERG (Vinberg and Koskelainen, 2010).

**Table 1:** Morphological parameters and estimation of end-on collecting area of rods in control and transgenic mice

Feature	Unit	Rho^+/+^ *Lyubarsky et al.(2004)	Rho^P23H/+^ (P30) *Estimated from data by Sakami et al. (2011, 2014); Chiang et al. (2015)	Rho^+/−^ (P30) *Estimated from data by Lem et al. (1999); Calvert et al. (2001)	Rho^+/−^ (P90) *Liang et al. (2004)
Rhodopsin protein expression	Percentage (relative to WT)	100%	∼50%	∼50%	∼50%
ONL thickness *Measured: Figures 1, 6	μm	55–60	35–40	55–60	50–55
OS length	μm	24	12	24	16
OS diameter	μm	1.4	1.4	1.4	1.1
OS volume	μm^3^	37	18.5	37	16
Rhodopsin concentration in OS	mm	3	3	1.5	3
ΔD	o.d. units/μm	0.019	0.019	half +/+ (*0.0095)	0.019
A_c_ single rod	μm^2^	0.87	0.55 (↓1.6-fold)	0.55 (↓1.6-fold)	0.41 (↓2-fold)

**Figure 1.**
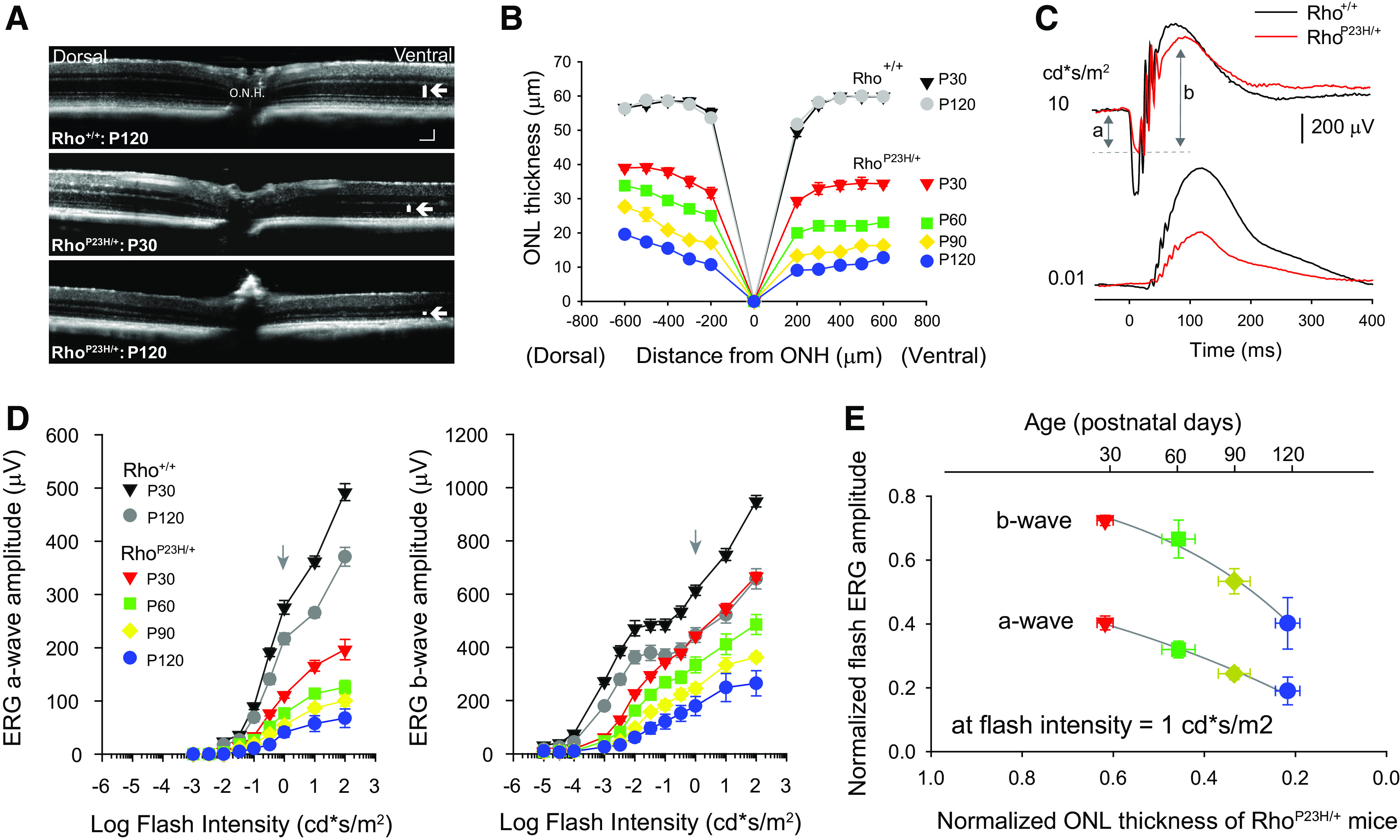
A gradual reduction in the ONL thickness and flash ERG responses of Rho^P23H/+^ mice. ***A***, Representative dorsal-ventral OCT images of Rho^+/+^ control and Rho^P23H/+^ retinas. Top, P120 Rho^+/+^ control retina. Middle, P30 Rho^P23H/+^ retina. Bottom, P120 Rho^P23H/+^ retina. Optic nerve head labeled ONH. White bars and arrows in ventral retina indicate approximate boundaries of the ONL. Scale bar: 50 μm. ***B***, Quantification of ONL thickness along the dorsal-ventral axis comparing Rho^+/+^ control and Rho^P23H/+^ mice. ***C***, Representative scotopic (dark-adapted) ERG waveforms of P30 Rho^+/+^ control (black traces) and Rho^P23H/+^ mice (red traces) in response to a bright flash (10  cd*s/m^2^, top traces) that elicits both an a-wave and a b-wave, and in response to a relatively dim flash (0.01  cd*s/m^2^, bottom traces) that elicits only a b-wave. Gray arrows indicate the magnitude of the a-and b-waves flash ERG response for Rho^P23H/+^ mice. Stimulus flashes were presented at 0 s. ***D***, Dark-adapted flash ERG intensity response functions depicting maximum (left) a-wave and (right) b-wave amplitudes as a function of flash intensity for Rho^+/+^ control and Rho^P23H/+^ mice. Arrows indicate responses to a 1 cd*s/m^2^ flash used to compare with ONL thickness in Figure 1*E*. Genotype and ages are indicated in the figure. Symbols represent mean ± SEM (in some cases, error bars are smaller than symbols), *n* = 9–10 mice. ***E***, Normalized a-wave and b-wave flash amplitudes of Rho^P23H/+^ mice plotted as a function of normalized ONL thickness measured at 500 mm from the ONH (average measurement of all four quadrants). Flash ERG amplitudes in the plot were measured in response to brief flash of 1 cd*s/m^2^. Flash ERG and ONL thickness measurements in Rho^P23H/+^ mice were normalized relative to those of age-matched Rho^+/+^ controls. Fits are exponential rise to maximum at normalized ONL thickness = 1.0 (*R*^2^ > 0.98). For all panels, genotype and ages are indicated in figure. Symbols represent mean ± SEM (in some cases, error bars are smaller than symbols); *n* = 4–5 mice.

All parameters were either measured in this study (ONL thickness from OCT measurements in Fig. 1) or from previously published studies (Rho^+/+^, Lyubarsky et al., 2004; Rho^P23H/+^, Sakami et al., 2011, 2014; Chiang et al., 2015; Rho^+/-^, Lem et al., 1999; Calvert et al., 2001; Liang et al., 2004). We then used the values listed here and Equation 7 in Materials and Methods to estimate the end-on collecting areas (A_c_) of Rho^+/+^ control, Rho^P23H/+^, and Rho^+/-^ mice.

The amplification factor (A) of the *ex vivo* ERG responses was determined by fitting the responses using a model (Eq. 1) as described by Lamb and Pugh, 1992.

(1) $$\frac{R\left(t\right)}{\phi }=\frac{1}{2}A{\left(t-t_{eff}\right)}^{2},$$

where R(t) is the response at a given time (t), ϕ is the stimulus intensity in R*/rod, A is the amplification factor representing the gain of the phototransduction cascade, t is time, and t_eff_ is the sum of all delays in activation. Analysis was performed only for responses to dim flash responses up to ∼100 R*/rod (Lamb and Pugh, 1992). Fits were performed at the early phase of the responses (t < 75 ms, normalized amplitude <0.2) and produced an *R*^2^ > 0.9 in all cases.

To measure isolated photoreceptor responses to flicker, a 75% contrast sinusoidal flickering stimulus of increasing mean illumination (−2 to 2 log cd/m^2^) was then presented at flicker frequencies of 3, 6, and 12 Hz. Each flicker trial was 1.6 s in duration for 3- and 6-Hz flicker stimulation and 0.8 s in duration for 12-Hz flicker stimulation. The response is an average of 30 trials for each condition. A Fourier transform analysis was performed to determine the fundamental magnitude (F0) of the response at each condition. Flicker ERG data were analyzed offline using SigmaPlot software.

We calibrated the stimuli of our *ex vivo* ERG system in terms of rates of photoisomerizations/rod/s following the procedure described by Vinberg et al. (2014). We found that, for control retinas, the sensitivities of normalized a- and b-wave dark adapted flash intensity-response functions measured *ex vivo* were approximately seven times more sensitive compared with those measured with *in vivo* conditions. Thus, for a given luminance value, the equivalent rate of photoisomerizations/rod/s for the *ex vivo* ERGs is estimated as the product of the conversion factor (7×) multiplied by the number of *in vivo* photoisomerizations/rod/s at that luminance. As described above, the *in vivo* rate for control retinas was estimated as per Lyubarsky et al. (2004), assuming an end-on rod collecting area = 1 μm^2^ and expressed in terms of R*/rod/s for wild type mice in all plots.

### Estimation of the steady state circulating current suppression

A saturating flash ERG protocol, similar to that described by Lyubarsky et al. (1999), was used to estimate the steady state circulating current suppression of rod photoreceptors (I_circ_). A series of *in vivo* flash ERG responses were obtained using a brief flash stimulus (4 ms in duration, ∼530 nm) of ∼1776  cd*s/m^2^ (producing ∼10^5^ R*/rod/s, bright enough to elicit a saturating a-wave amplitude; Lyubarsky and Pugh, 1996). The flash stimulus was presented first in dark-adapted conditions and then at increasing levels of mean background illumination (2 to 2 log cd/m^2^). Increasing the background illumination reduces the circulating current and thus, the a-wave response to the saturating flash decreased in amplitude with increasing background illumination, reflecting a smaller reduction in circulating current by the flash. To quantify the suppression of circulating current evoked by background illumination (I_circ_), the following analysis was applied (Lyubarsky et al., 1999):

(2) $${\text{I}}_{\text{circ}}=\text{ 1 }–\left(\text{R}/{\text{R}}_{\text{dark}}\right),$$

**Table 2:** Model parameters

Parameter	Symbol	Rho^+/+^ (Rho^P23H/+^ control)	Rho^P23H/+^	Rho^+/+^ (Rho^+/−^ control)	Rho^+/−^
Scaling constant*	K0	750	675	1100	990
Relative ONL thickness	f_ONL_	1	0.6	1	1
Dominant time constant	τ (s)	0.135	0.055	0.138	0.068
Irradiance at half maximal output	EC_50_ (ph/μm^2^/s)	300	300	300	300
Rod length	L (μm)	24	12	24	24
Rod diameter	d (μm)	1.4	1.4	1.4	1.4
Quantum efficiency	q	0.63	0.63	0.63	0.63
Funneling	fp	1.2	1.2	.1.2	1.2
Pigment optical density	ΔD (od units/μm)	0.019	0.019	0.019	0.0095
*K0 @12Hz = 0.6×K0					

Model parameters for the model fits depicted in Figure 5. All parameters were measured in this study (ONL thickness, Fig. 1; τ, Fig. 7) or obtained from the literature (see Table 1).

where R is the a-wave response to a saturating flash at a given background illumination level and R_dark_ is the a-wave response to a saturating flash in darkness. These data were fit with a hyperbolic saturation function to determine the half-saturating intensity (I_50CS_) for each transgenic mouse line and respective controls.

### Model equations

A sinusoidally modulated flicker input is given by the following:

(3) I(t)=I[1 + Csin(wt)],

where *C* is the modulation contrast, *I* is the mean retinal irradiance (in photons/rod/s) originating from a monochromatic green (530 nm) LED light source, *w = 2πf_0_*, is the flicker in radians, and *f_0_* is the flicker frequency in Hertz. The response recorded with the *in vitro* transretinal ERG was modeled in terms of a linear-nonlinear system, where the flicker response is given by Umino et al. 2019 (their Eq. 14) and modified to account for differences in rod collecting areas and ONL thickness in Rho^P23H/+^, Rho^+/−^ and Rho^+/+^ retinas:

(4) $$y_{ss}(t)=K\frac{AI+\beta AIsin\left(wt+\varphi \right)}{AI+\beta AIsin\left(wt+\varphi \right)+\gamma EC_{50}},$$

where

(5) $$\beta =\frac{aC}{\sqrt{a^{2}+w^{2}}}$$

is the attenuation factor of the first order linear filter, τ is its time constant, $a=\tau^{-1}$ is the rate, and

(6) $$\gamma ={\left(h_{0}\tau \right)}^{-1}$$

is the inverse of response integration area.

*A* is the end-on rod collecting area given by Lyubarsky et al. (2004):

(7) $$A\left(\lambda \right)=f\text{π}\frac{d^{2}}{4}\left[1-10^{-\text{Δ}D\left(\lambda \right)L}\right]q,$$

where *f = 1.3* is the light funneling by the inner segment, *q* = 0.63 is the quantal efficiency*, L* is the rod outer segment length, *d* is its diameter, and *ΔD(λ)* (in od/μm) is the specific axial density. We calculated the end-on collecting area of Rho*^P23H/+^*and Rho^+/−^ rods at P30 using Equation 7 and the parameter values listed in Table 1. *K* is a scaling factor that depends on the extracellular resistance and the number of rods in the retina, which we assume is proportional to f_ONL_, the fractional thickness of the ONL (relative to that of control retinas). Hence,

(8) $$K=K_{0}f_{ONL}.$$

Hence, the maximal magnitude of the flicker response (Umino et al., 2019; their Eq. 13) can be expressed as a function of retinal irradiance (*I*), collecting area (*A*) and response integration time (*δ*).

(9) $$\Delta y_{ss}=\frac{K\delta \beta AEC_{50}I}{\left(1-\beta^{2}\right)\delta^{2}A^{2}I^{2}+2\delta AEC_{50}I+EC_{50}^{2}},$$

where $\delta =\gamma^{-1}=h_{0}\tau$ and *K* is same as in Equation 8.

At steady state, without contrast flicker, C = 0, Equation 4 simplifies to:

(10) $$y_{ss}=\frac{I}{I+\gamma \frac{EC_{50}}{A}}.$$

From Equation 10, we derive an expression for the irradiance level *I_50%_*that elicits a half maximal response in steady state $\left(y_{ss}=0.5\right)$,

(11) $$I_{50\%}=\gamma \frac{EC_{50}}{A}.$$

Assuming that the magnitude of the response to steady lights is proportional to the suppression of the rod circulating currents (I_circ_) by those steady lights, and that transgenic (*T*) and control (*C*) mouse lines have the same EC_50_ value (Umino et al., 2019); then, from Equation 11, we derive the following relations:

(12) $$EC_{50}=A_{C}\frac{I_{50\%}^{c}}{\gamma_{C}}=A_{T}\frac{I_{50\%}^{T}}{\gamma_{T}}$$

(13) $$\frac{I_{50\%}^{T}}{I_{50\%}^{c}}=\frac{A_{C}}{A_{T}}\frac{\gamma_{T}}{\gamma_{C}}=\frac{A_{C}}{A_{T}}\frac{h_{0C}}{h_{0T}}\frac{\tau_{C}}{\tau_{T}}.$$

Equation 13 indicates that the ratio of the *I_50%_* values in transgenic and control retinas is proportional to the ratios of the response kinetics, the amplification factors, and the collecting areas in control and transgenic retinas. As a first approximation we assume that (1) the kinetics are proportional to the τ_D_ and do not change with state of light adaptation or bleaching; and (2) that transgenic and control retinas have matching amplification factors (h_o_). Collecting areas (A_c_ and A_T_) are estimated using previously published values (see Table 2).

With knowledge of the collecting areas and kinetics (Table 2), we can rearrange Equation 13 to estimate the steady light levels (*I^T^*) that suppress the circulating currents in transgenic retinas:

(14) $$I^{T}=\frac{A_{C}}{A_{T}}\frac{h_{0C}}{h_{0T}}\frac{\tau_{C}}{\tau_{T}}I^{C}.$$

### Quantification and statistical analysis

Quantification of ONL thickness measurements of Rho^+/+^, Rho^P23H/+^, and Rho^+/−^ mice was performed using a two-way repeated measures analysis of variance (two-way RM ANOVA) with nominal factors being genotype (or age) and retinal location. If significant interactions were detected, Holm-Sidak’s procedure for pairwise multiple comparisons was performed to determine where the significant interactions occurred. A similar analysis was applied to quantify flash ERG a- and b- wave amplitudes (nominal factors being genotype (or age) and flash intensity), flicker ERG amplitude, retinal TCS, and behavioral TCS of Rho^+/+^, Rho^P23H/+^, and Rho^+/−^ mice (nominal factors being genotype and temporal frequency, or mean illumination). One-way ANOVAs were performed to quantify the changes in ONL thickness of Rho^P23H/+^ mice with age in each of the four retinal quadrants and the τ_D_ of recovery of Rho^+/+^, Rho^P23H/+^, and Rho^+/−^ mice. When necessary, logarithmic transformations of the data were performed before statistical analysis to fulfil normality and equal variance requirements for the ANOVAs. To determine whether our model could explain the enhanced photoresponses of Rho^P23H/+^ and Rho^+/−^ retinas recorded with the transretinal ERG, we applied maximum likelihood estimation (Millar, 2011). The responses to 3-, 6-, and 12-Hz flicker were evaluated independently and the corresponding values of h_0_, the single free variable in the model, and fit coefficients are listed in Table 3. Data analysis was performed with SigmaStat software (Systat Software). In all plots, filled symbols display mean ± SEM of the data and in some cases the error bars are smaller than the symbols. Numbers of mice and *p* values are indicated in the figure legends.

**Table 3:** Model variable and fit coefficients

Frequency	Rho^P23H/+^			Rho^+/−^		
	h_0_	*R*^2^	RMSE	h_0_	*R*^2^	RMSE
3 Hz	6.3	0.45	8.63	6.9	0.62	11.7
6 Hz	3.3	0.98	1.20	3.8	0.88	5.9
12 Hz	1.5	0.77	1.76	1.0	0.77	1.82

Model variables and fit coefficients for fits depicted in Figure 5. The applied model is expressed in Materials and Methods, Equation 13. h_0_ is the only variable in the model. All other parameters in the model are measured and listed in Table 2. *R*^2^ is the coefficient of determination of the fit, and RMSE is the root mean square error representing the SD of the residuals.

## Results

### A gradual reduction in ONL thickness and flash ERG responses in Rho^P23H/+^ mice

Rho^P23H/+^ mice exhibit a rapid rate of photoreceptor degeneration between P15 and P30 followed by a more gradual rate of degeneration at later time points (Sakami et al., 2011; Chiang et al., 2015). The rate of degeneration in animal models with this mutation depends critically on housing illumination levels and daily light exposure (Naash et al., 1996; Organisciak et al., 2003; Walsh et al., 2004; Paskowitz et al., 2006; Tam and Moritz, 2007; Orlans et al., 2019). We used OCT to obtain images of Rho^+/+^ and Rho^P23H/+^ retinas *in vivo* and assessed the degree of photoreceptor loss by measuring the thickness of the ONL along the dorsal-ventral (Fig. 1*A*,*B*) and nasal-temporal (data not shown) axes. The ONL thickness of Rho^+/+^ control mice was relatively constant (55–60 μm) across all retinal locations and did not change significantly from P30 to P120 (*p* = 0.786 for dorsal-ventral axis, *p* = 0.352 for nasal-temporal axis, two-way RM ANOVA; Fig. 1*B*). In contrast, the ONL thickness of P30 Rho^P23H/+^ mice was reduced by 30% – 40% compared with controls at all retinal locations (*p* < 0.001, two-way RM ANOVA; Fig. 1*B*) and decreased gradually with age (Fig. 1*A*,*B*), as described previously (Sakami et al., 2011).

We next examined the relationship between ONL thickness and scotopic flash ERG sensitivity of Rho^P23H/+^ mice. Both the photoreceptor driven ERG a-waves and the bipolar cell driven b-waves of Rho^P23H/+^ mice were significantly reduced in amplitude compared with those of Rho^+/+^ controls (*p* < 0.001, two-way RM ANOVA; Fig. 1*C*,*D*), in agreement with previous reports (Sakami et al., 2011; Leinonen et al., 2020). We compared flash ERG responses at 1  cd*s/m^2^, the dimmest flash that elicited robust a- and b-wave responses in Rho^P23H/+^ mice at all the time points of our study (Fig. 1*D*, arrows), with the ONL thickness. ONL thickness was measured at 500 μm from the nerve head and averaged across all quadrants. ERG response amplitudes and ONL thickness values were normalized relative to those of age-matched control mice to account for losses that can be attributed to natural aging (Li et al., 2001; Gresh et al., 2003; Kolesnikov et al., 2010). Plots of normalized ERG responses versus the normalized ONL thickness (Fig. 1*E*) show two features: (1) a larger initial reduction in a-wave responses (∼60%) compared with that of b-wave responses (∼30%), suggesting a relative preservation of bipolar cell driven b-wave responses in Rho^P23H/+^ mice, in agreement with a recent report (Leinonen et al., 2020), and (2) a gradual reduction in flash ERG signals concomitant with the reduction of ONL thickness.

### A frequency-dependent enhancement in the flicker ERG of Rho^P23H/+^ mice

**Figure 2.**
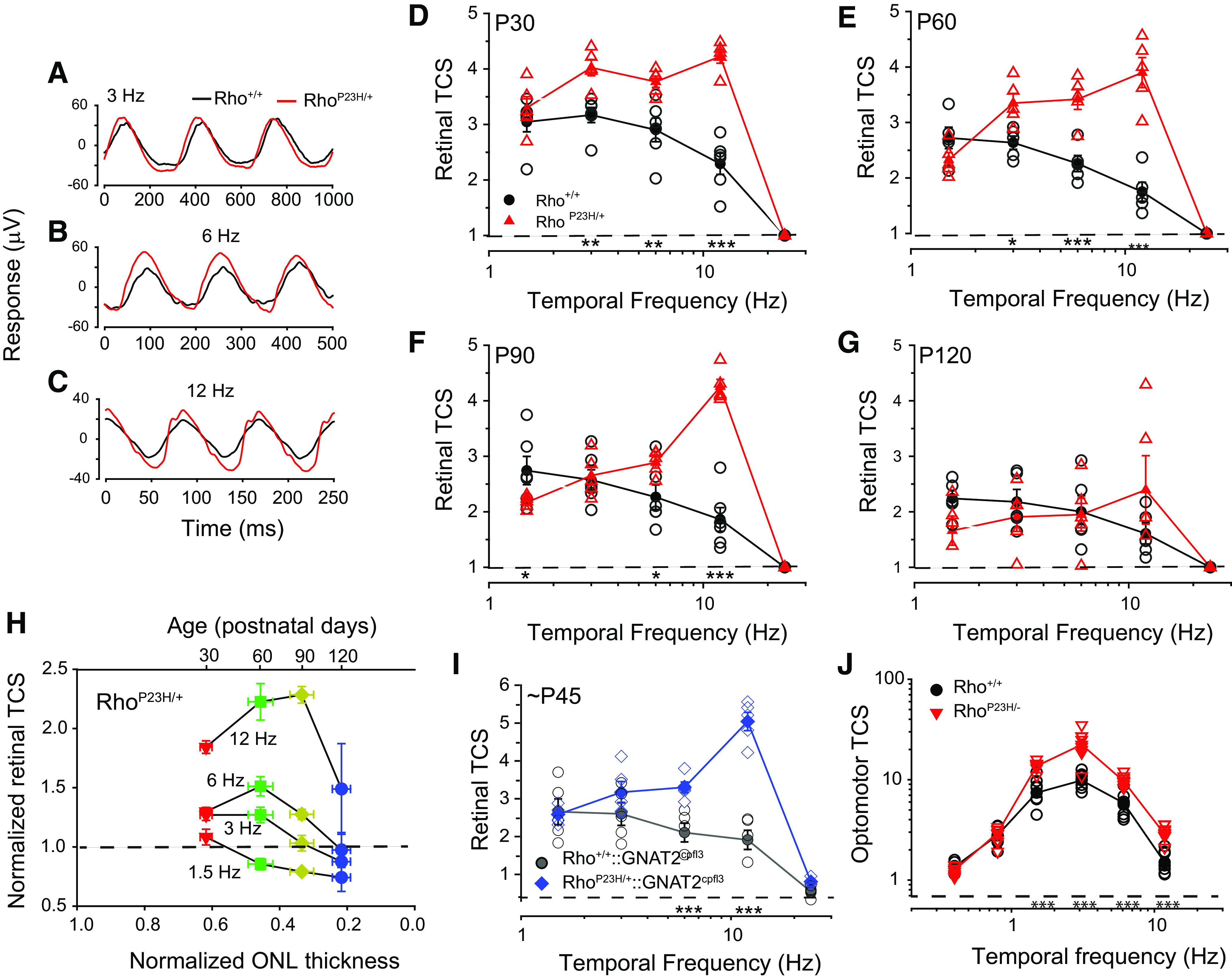
Rho^P23H/+^ mice exhibit a frequency-dependent enhancement in flicker ERG sensitivity compared with controls. ***A–C***, Averaged P30 flicker ERG responses of Rho^+/+^ (black traces) and Rho^P23H/+^ mice (red traces). All traces are responses to a 75% contrast sinusoidal flickering stimulus with a mean background illumination of 2 cd/m^2^ (∼1250 R*/rod/s): (***A***) 3 Hz, (***B***) 6 Hz, (***C***) 12 Hz. ***D–G***, Retinal TCSFs of Rho^+/+^ control (black circles) and Rho^P23H/+^ mice (red triangles) at a mean background illumination of 2 cd/m^2^ (∼1250 R*/rod/s). TCSF plots depict retinal contrast sensitivity as a function of temporal frequency at (***D***) P30, (***E***) P60, (***F***) P90, (***G***) P120. For all panels, filled symbols represent mean ± SEM (in some cases, error bars are smaller than symbols), and open symbols represent data for individual mice. *N* = 5–6 mice for each genotype and age. Statistical analysis: two-way RM ANOVA; *p < 0.05, **p < 0.01, ***p < 0.001. ***H***, Retinal TCS of Rho^P23H/+^ mice increases as ONL thickness decreases. Retinal TCS of Rho^P23H/+^ mice as a function of ONL thickness (average measurement of all four quadrants) and age. Rho^P23H/+^ measurements are normalized relative to age-matched Rho^+/+^ control measurements. Dotted line represents a value of 1 where Rho^P23H/+^ measurements are equal to those of controls. Filled symbol represent mean ± SEM, *n* = 5–6 mice for each genotype and age. ***I***, Enhanced TCS of Rho^P23H/+^ mice is not mediated by cone overcompensation or altered rod-cone interactions. TCSFs depicting retinal TCS as a function of temporal frequency of Rho^+/+^::GNAT2^cpfl3/cpfl3^ control (gray circles) and Rho^P23H/+^:GNAT2^cpfl3/cpfl3^ mice (blue diamonds) at a mean background illumination of 2 cd/m^2^ (∼1250 R*/rod/s). Filled symbols represent mean ± SEM (in some cases, error bars are smaller than symbols), and open symbols represent responses of individual mice. *N* = 5 mice for each genotype. Statistical analysis: two-way RM ANOVA; ***p < 0.001. ***J***, Enhanced optomotor contrast sensitivity in Rho^P23H/+^ mice. TCSFs measured with an optomotor behavior assay at P30–P45. Plots compare TCS of Rho^+/+^ control litter mates and Rho^P23H/+^ mice. Mean background illumination was 70 cd/m^2^ (∼1500 R*/rod/s), and spatial frequency was set to 0.236 cycles/°. Filled symbols represent mean ± SEM (in some cases, error bars are smaller than symbols), and open symbols represent responses of individual mice. *N* = 7–8 mice for each frequency and genotype. TCS was determined by averaging responses over 4 days. Statistical analysis: two-way RM ANOVA; ***p < 0.001.

We compared flicker ERG responses of Rho^+/+^ and Rho^P23H/+^ mice at P30, the time point at which retinal degeneration in Rho^P23H/+^ mice is least severe (Fig. 1). We chose a mesopic illumination rather than a rod isolating scotopic illumination because, in mouse, rod photoresponse kinetics control TCS under mesopic (Umino et al., 2019), but not under scotopic conditions (Umino et al., 2012, 2019; Peinado Allina et al., 2017). We measured flicker ERG responses to a sinusoidal stimulus (mean illumination: 2 cd/m^2^, 75% contrast) at multiple temporal frequencies (1.5–24 Hz; Fig. 2). The responses of Rho^+/+^ control and Rho^P23H/+^ mice to a low flicker frequency of 3 Hz had a similar waveform, but with a slight increase in amplitude and an advance in the phase of the response of Rho^P23H/+^ mice (Fig. 2*A*). By contrast, the responses of Rho^P23H/+^ mice to intermediate frequencies of 6 and 12 Hz were larger than those of Rho^+/+^ control mice (Fig. 2*B*,*C*).

We measured the retinal TCSFs (see Materials and Methods) to determine the frequency-dependent differences in mesopic flicker ERG responses of P30 Rho^+/+^ and Rho^P23H/+^ mice (Fig. 2*D*). All measurements were performed at 2 cd/m^2^, the light level at which enhanced flicker ERG responses were observed in P30 Rho^P23H/+^ mice (Fig. 2*A–C*). At P30, the retinal TCSFs of Rho^+/+^ mice were low pass shape while those of Rho^P23H/+^ mice exhibited a slight attenuation in the responses to low frequencies (Fig. 2*D*). Rho^P23H/+^ mice exhibited enhanced TCS compared with controls at intermediate temporal frequencies of 3, 6, and 12 Hz (*p* < 0.001, two-way RM ANOVA; Fig. 2*D*) but not at the low (1.5 Hz) and high (24 Hz) ends of the frequency spectrum (*p* = 0.618 and 0.936 for 1.5 and 24 Hz, respectively, two-way RM ANOVA).

**Figure 3.**
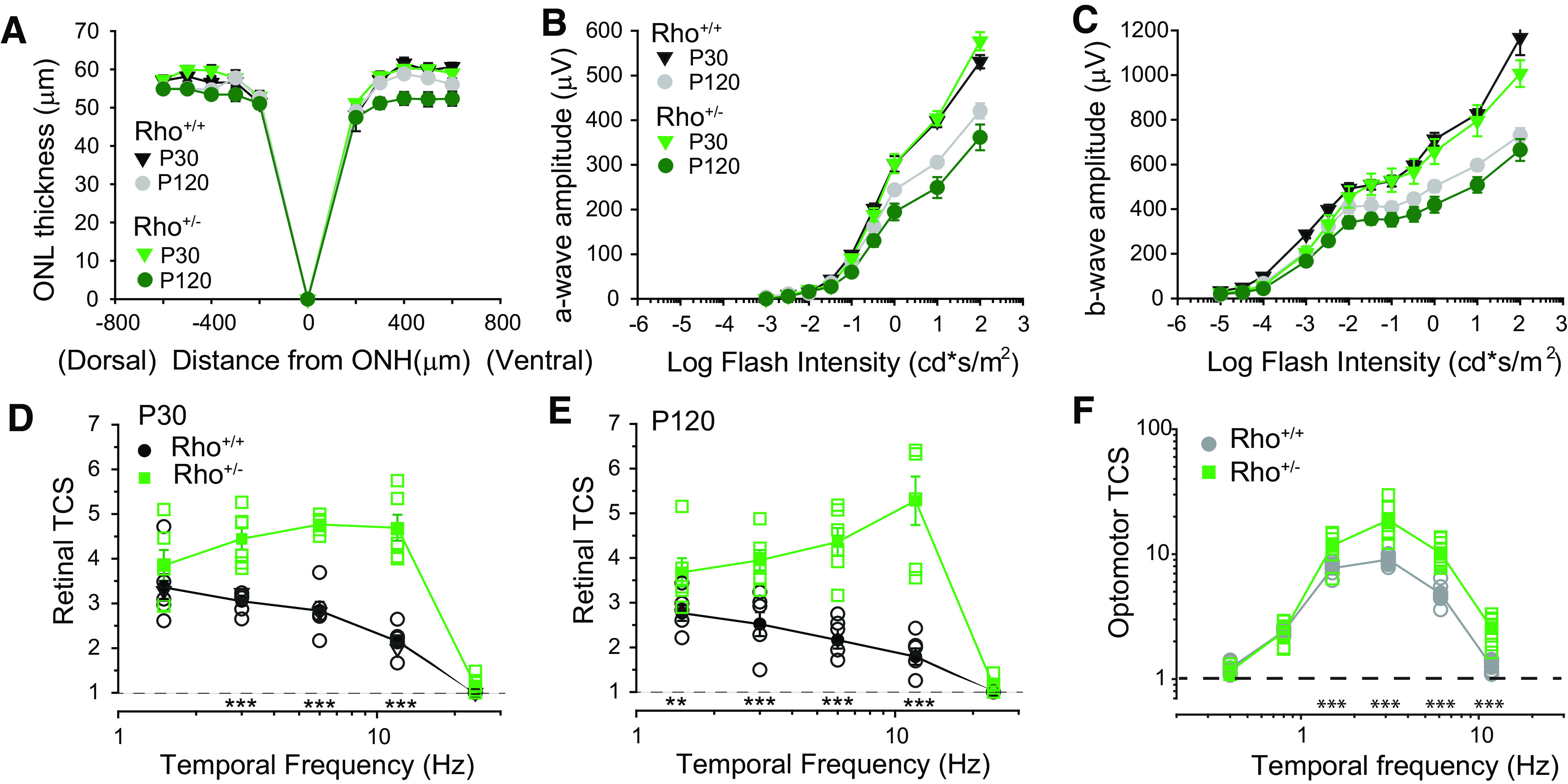
Enhanced retinal TCS precedes retinal degeneration in Rho^+/−^ mice. ***A***, Minimal age-dependent reduction in ONL thickness measured from OCT images. Quantification of ONL thickness along the dorsal-ventral axis comparing Rho^+/+^ control siblings and Rho^+/−^ mice. Genotype and ages indicated in plot. *N* = 4–5 mice. ***B***, ***C***, Minimal age-dependent reduction in flash ERG amplitude in Rho^+/−^ mice. Dark-adapted flash ERG intensity response functions depicting maximum (***B***) a-wave and (***C***) b-wave amplitudes as a function of flash intensity for Rho^+/+^ control and Rho^+/−^ mice. *N* = 9–10 mice. ***D***, ***E***, Retinal TCSFs of Rho^+/+^ control and Rho^+/−^ mice measured by flicker ERG depicting TCS as a function of temporal frequency at (***D***) P30 and (***E***) P120. For all panels, filled symbols represent mean ± SEM (in some cases, error bars are smaller than symbols), and open symbols represent responses of individual mice, *N* = 5–6 mice. Statistical analysis: two-way RM ANOVA; *p < 0.05, **p < 0.01, ***p < 0.001. ***F***, Enhanced optomotor contrast sensitivity in Rho^+/−^ mice. Optomotor contrast sensitivity functions at P30–P45. Plots compare TCS of Rho^+/+^ control litter mates and Rho^+/−^ mice. Mean background illumination was 70 cd/m^2^ (∼1500 R*/rod/s), and spatial frequency was set to 0.236 cycles/°. Filled symbols represent mean ± SEM (in some cases, error bars are smaller than symbols), and open symbols represent responses of individual mice. *N* = 7–8 mice for each time point and genotype. TCS was determined by averaging responses over 4 d. Statistical analysis: two-way RM ANOVA; ***p < 0.001.

**Figure 4.**
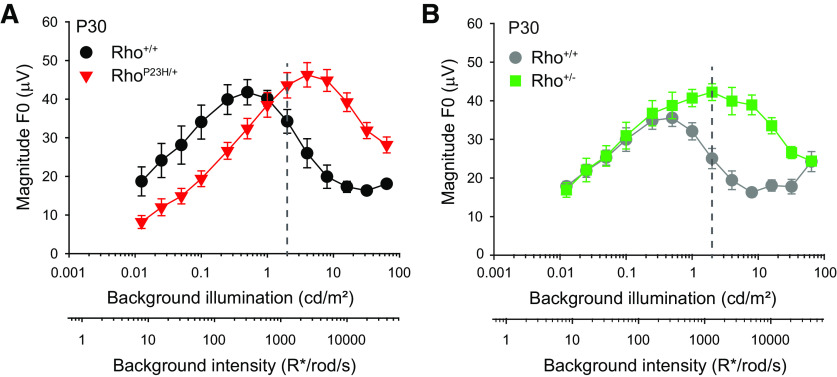
Rho^P23H/+^ and Rho^+/−^ mice exhibit distinct illumination-dependent enhancement of their flicker ERG magnitude functions. ***A***, ***B***, Magnitude of the fundamental frequency (F0) in the flicker ERG response to 6-Hz flicker with 75% contrast presented at multiple mean background illumination levels. Plots show the responses of (***A***) Rho^+/+^ control sibling (black circles) and Rho^P23H/+^ mice (red triangles) at P30 and (***B***) Rho^+/+^ control sibling (gray circles) and Rho^+/−^ mice (green squares) at P30. Dashed lines indicate the mean illumination level of 2 cd/m^2^ (∼1250 R*/rod/s) used in Figures 2, 3. Symbols represent mean ± SEM (in some cases, error bars are smaller than symbols), *n* = 4–6 mice. Statistical analysis: two-way RM ANOVA. In ***A***, *p* > 0.05 at 1 cd/m^2^, *p* < 0.05 at all other illumination levels. In ***B***, *p* > 0.05 at illumination levels <1 cd/m^2^, *p* < 0.05 at all other illumination levels. Intensity levels are expressed in terms of the expected photoisomerization rate (R*/rod/s) for WT rods.

### Early loss in retinal TCS of Rho^P23H/+^ mice to low temporal frequencies

We determined how the retinal TCSFs of Rho^P23H/+^ mice changed as degeneration progressed. By P60, retinal TCS of Rho^P23H/+^ mice remained strong and significantly higher than that of control mice at intermediate temporal frequencies of 3, 6, and 12 Hz (*p* < 0.01, two-way RM ANOVA; Fig. 2*E*). This strong and enhanced TCS occurred despite the additional ∼10% – 15% decline in ONL thickness and flash ERG responses observed in Rho^P23H/+^ mice during this time period (Fig. 1). By P90, retinal TCS of Rho^P23H/+^ mice exhibited considerable loss in sensitivity to 1.5, 3, and 6 Hz but not in the responses to 12 Hz (1.5 Hz: *p* = 0.028; 3 Hz: *p* = 0.742; 6 Hz: *p* = 0.018; 12 Hz: *p* < 0.001, two-way RM ANOVA; Fig. 2*F*). By P120, we no longer observed any significant differences in the TCS of Rho^P23H/+^ mice compared with controls (*p* > 0.05, two-way RM ANOVA; Fig. 2*G*). The retinal TCSFs of Rho^+/+^ control mice retained their characteristic low-pass shape but demonstrated a uniform, mild reduction in sensitivity to all temporal frequencies. The losses in sensitivity followed the time course of the age-dependent reduction in flash ERG magnitude that we observed in control mice (Fig. 1). By contrast, retinal TCSFs in Rho^P23H/+^ mice demonstrated an early, gradual loss in sensitivity to low flicker frequencies (1.5, 3, and 6 Hz) that became a more sharply tuned bandpass with a peak at 12 Hz by P90.

To relate TCS to the degree of retinal degeneration we plotted normalized retinal TCS of Rho^P23H/+^ mice as a function of normalized ONL thickness (Fig. 2*H*). We normalized the retinal TCS and ONL thickness of Rho^P23H/+^ mice relative to that of age-matched control mice to account for the natural losses associated with aging. The normalized retinal TCS to 1.5-Hz flicker was similar in P30 Rho^P23H/+^ and control mice (normalized retinal TCS ∼1) and declined gradually with age and ONL thickness. In response to 3-Hz flicker, TCS of P30 Rho^P23H/+^ mice was initially enhanced by ∼1.25-fold compared with controls and, surprisingly, remained relatively stable at P60 although the ONL thickness was reduced by 55% compared with controls. Beyond that point, TCS to 3-Hz flicker decreased gradually with increasing age and further reduction in ONL thickness. Lastly, in response to 6- and 12-Hz flicker, TCS of P30 Rho^P23H/+^ mice was enhanced by ∼1.25-fold and ∼1.75-fold compared with controls, respectively. Remarkably, TCS increased further, to ∼1.5-fold and ∼2.25-fold by P60 when the ONL thickness was reduced by 55% compared with controls. In the case of 12-Hz flicker, TCS continued to increase reaching a normalized enhancement of ∼2.3-fold at P90, when the ONL thickness was reduced by 70–75% compared with controls. Thereafter, retinal TCS declined sharply and by P120, when the ONL thickness is reduced by almost 80% compared with controls, TCS to 12-Hz flicker remained ∼1.5-fold higher than controls. These results demonstrate a progressive, frequency-dependent enhancement in normalized retinal TCS as the retina degenerates. The point at which normalized TCS declines depends on temporal frequency, but can be as late as P90, when the ONL thickness is reduced by 70–75%.

### Cone photoresponses do not mediate the increase in TCS in Rho^P23H/+^ mice

A possible explanation for the increased TCS in Rho^P23H/+^ mice is cone overcompensation or altered rod-cone interactions (Okado et al., 2017), producing larger flicker ERG responses. However, Rho^P23H/+^ mice on a transgenic background with disrupted cone responses (GNAT2^cpfl3/cpfl3^ mice; Chang et al., 2006; Allen et al., 2010; Brown et al., 2011; Pasquale et al., 2020) also exhibited increased retinal TCS compared with controls at P30 (Fig. 2*I*). Note that the residual cone activity in GNAT2^cpfl3/cpfl3^ mice does not mask rod responses under the mesopic illumination conditions (∼1500 R*/rod/s) used in this study (Umino et al., 2019; Pasquale et al., 2020). These results show that cone photoresponses do not play a dominant role in enhancing TCS in Rho^P23H/+^ mice.

### Behavioral TCS is enhanced in Rho^P23H/+^ mice

We determined whether the increases in retinal TCS of Rho^P23H/+^ mice translates into enhanced behavioral TCS. The early retinal degeneration present in Rho^P23H/+^ mice prevented us from training mice sufficiently to perform operant visual tasks. Therefore, we measured behavioral TCS using the optomotor reflex assay (Prusky et al., 2004; Umino et al., 2008) in P30–P45 Rho^+/+^ control and Rho^P23H/+^ mice (Fig. 2*J*). The illumination level of the optomotor assay (70 cd/m^2^ eliciting ∼1500 R*/rod/s) was chosen to approximate the level of retinal illumination of the flicker ERGs (1250 R*/rod/s; Fig. 2). Other stimulus parameters are listed in the figure legend. Rho^P23H/+^ mice exhibited a frequency-dependent enhancement in behavioral TCS compared with controls (significant genotype and genotype × frequency interactions: *p* < 0.001, two-way RM ANOVA; Fig. 2*J*). Optomotor TCS to 1.5, 3, 6, and 12 Hz was ∼2-fold higher in Rho^P23H/+^ mice than in control mice (*p* < 0.001). In contrast, optomotor TCS at the low end of the frequency spectrum (0.4 and 0.8 Hz) was not significantly changed between Rho^P23H/+^ and controls (*p* = 0.478 and *p* = 0.971, respectively), in line with recent findings (Leinonen et al., 2020). These enhanced optomotor responses to intermediate and high frequencies are similar to the enhanced retinal TCS measured with flicker ERGs (Fig. 2*D*). Thus, enhanced retinal activity detected with the flicker ERG translates into an improvement in a visual reflex behavior.

### Enhanced retinal TCS without retinal degeneration in Rho^+/−^ mice

Enhanced retinal TCS in Rho^P23H/+^ mice could arise from degenerative/remodeling processes (Leinonen et al., 2020). To examine TCS in the absence of retinal degeneration, we measured retinal TCS in a rhodopsin heterozygous mouse model (Rho^+/−^ mice; Lem et al., 1999), which has reduced rod collecting areas (see Table 1) and accelerated rod recovery kinetics but no degeneration before ∼P90–P120 (Fig. 3*A–C*; Lem et al., 1999; Calvert et al., 2001; Liang et al., 2004). Similar to Rho^P23H/+^ retinas, retinal TCS of Rho^+/−^ mice exhibited a slight bandpass shape, peaking at 6–12 Hz (Fig. 3*D*) but were larger compared with controls at intermediate flicker frequencies (3, 6, and 12 Hz, *p* < 0.001, two-way RM ANOVA; Fig. 3*D*), but not at 1.5 or 24 Hz (*p* = 0.102 and *p* = 0.073, respectively; two-way RM ANOVA). The retinal TCSFs of P30 Rho^+/−^ mice are similar in shape but slightly larger in magnitude than those of P30 Rho^P23H/+^ mice (compare to Fig. 2*D*). At P120, retinal TCS of Rho^+/−^ mice remained significantly higher than that of control mice (1.5 Hz, *p* = 0.02; all other frequencies, *p* < 0.001, two-way RM ANOVA; Fig. 3*E*). In fact, retinal TCS did not change as mice aged from P30 to P120. These results are in contrast to those in P120 Rho^P23H/+^ mice, where there was a prominent age-dependent decrease in retinal TCS coincident with the severe degeneration by this age (Fig. 2*G*). Similar to Rho^P23H/+^ mice, optomotor TCS to 1.5, 3, 6, and 12 Hz was ∼2-fold higher in Rho^+/−^ mice than in control mice (*p* < 0.001, two-way RM ANOVA; Fig. 3*F*). Together, these results demonstrate that increased retinal and optomotor TCS occurs in the absence of retinal degeneration in Rho^+/−^ mice and may be related to changes in collecting area (Table 1) and/or rod accelerated response kinetics (Calvert et al., 2001).

### Flicker ERG responses of Rho^P23H/+^ mice are enhanced at high luminance levels but reduced at low luminance levels

6HZ flicker elicits enhanced responses in Rho^P23H/+^ mice when presented at 2 cd/m^2^ (Fig. 2). We compared the fundamental magnitude of the ERG responses to 6-Hz flicker over a range of background intensities spanning the scotopic to mesopic range in P30 Rho^+/+^ and Rho^P23H/+^mice (Fig. 4*A*). In these conditions, the intensity-magnitude response functions of both control and Rho^P23H/+^ mice exhibited non-monotonic relationships with background illumination. In each case, the fundamental magnitude of the flicker ERG responses grew steadily with background illumination, reached a peak, and then declined gradually with further increases in background illumination (Fig. 4*A*). However, compared with control mice, the intensity-response curves of Rho^P23H/+^ mice were displaced rightward (responses peak in amplitude at illumination levels of 0.5 and 4 cd/m^2^, respectively) and slightly upward (peak response magnitudes are ∼40 and ∼50 μV, respectively). These responses demonstrate two well defined illumination ranges: (1) <1 cd/m^2^, where the absolute magnitudes of Rho^P23H/+^ responses are below control responses and (2) >1 cd/m^2^, where Rho^P23H/+^ responses are above control responses (*p* < 0.05, two-way RM ANOVA; Fig. 4*A*). Hence, the 6-Hz flicker responses of P30 Rho^P23H/+^ mice are enhanced compared with controls only at relatively bright, mesopic background illumination levels (>1 cd/m^2^) but are attenuated at lower illumination levels.

The right shift of the magnitude functions in Rho^P23H/+^ rods could result from a shift in sensitivity because of the lower amounts of rhodopsin in their rod outer segments relative to control rods (Sakami et al., 2011, 2014; Chiang et al., 2015). To investigate this possibility, we compared ERG responses to 6-Hz flicker of P30 Rho^+/+^ and Rho^+/−^ mice. Rho^+/−^ mice exhibit reduced rhodopsin expression in their outer segments (Table 1) but without any overt retinal degeneration at this time point (Fig. 3*A*). Similar to Rho^P23H/+^ retinas, the responses of Rho^+/−^ retinas were significantly higher than controls at background intensities >1 cd/m^2^ (Fig. 4*B*). However, in contrast to Rho^P23H/+^ retinas, the responses of Rho^+/−^ and control retinas were similar at background intensities <1 cd/m^2^ (Fig. 4*B*). Given that rods in both Rho^P23H/+^ and Rho^+/−^ have similar collecting areas (Table 1), we conclude that the differences in the magnitude response functions of Rho^P23H/+^ and Rho^+/−^ retinas (relative to control) cannot be explained exclusively in terms of changes in their collecting areas. Other factors, such as changes caused by degeneration or in rod photoresponse kinetics may determine magnitude response alterations.

**Figure 5.**
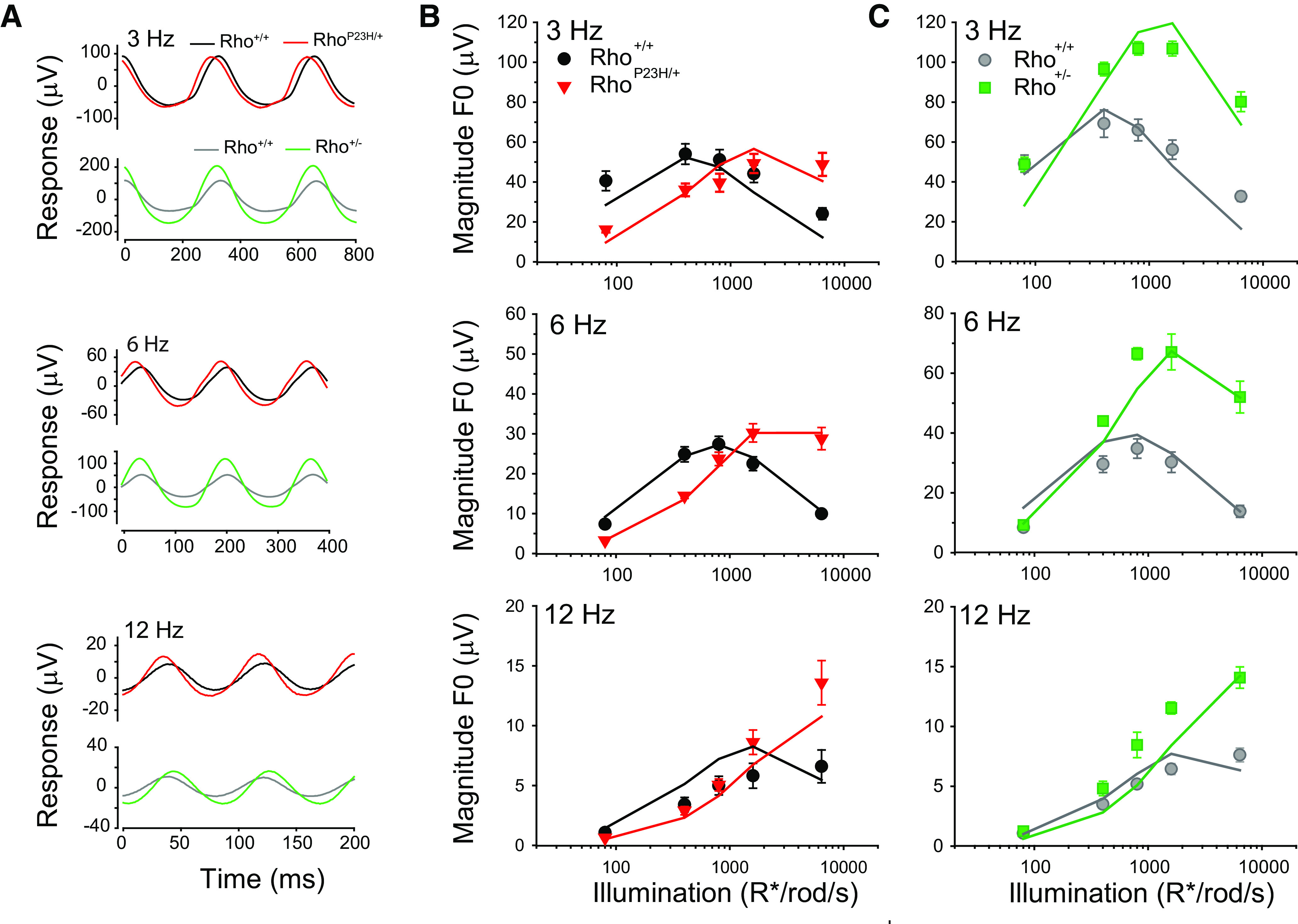
Enhanced photoreceptor responses in Rho^P23H/+^ and Rho^+/−^ mice compared with control mice. ***A***, Averaged photoreceptor isolated *ex vivo* ERG responses to 3-, 6-, and 12-Hz flicker comparing P30 Rho^+/+^ control responses and (top) Rho^P23H/+^ responses or (bottom) Rho^+/−^ responses. All traces are responses to 75% contrast sinusoidal flickering stimulus with a mean background illumination of 0.3 cd/m^2^ (∼1500 R*/rod/s). ***B***, ***C***, Fundamental magnitude of 3, 6, and 12 Hz, 75% contrast photoreceptor isolated *ex vivo* flicker ERG responses as a function of background illuminations comparing P30 Rho^P23H/+^ responses (***B***) or Rho^+/−^ responses (***C***) and their littermate Rho^+/+^ control responses. Filled symbols represent measured data mean ± SEM (in some cases, error bars are smaller than symbols). *N* = 3–4 retinas from at least two mice. Continuous lines represent model fits using Equation 9 (see text for details). Illumination levels are expressed in terms of the expected photoisomerization rate (R*/rod/s) for WT rods.

To determine whether the increase in TCS can be traced to the contrast responses of rod photoreceptors, we measured isolated photoreceptor responses of P30 Rho^P23H/+^, Rho^+/−^, and their littermate control mice, in response to flickering stimulus using an *ex vivo* transretinal ERG preparation (Sakami et al., 2014; Vinberg et al., 2014; Vinberg and Kefalov, 2015; see Materials and Methods). We found enhanced and phase shifted responses of Rho^P23H/+^ and Rho^+/−^ photoreceptors relative to controls in response to 75% contrast, 3-, 6-, and 12-Hz flickers presented at a background level eliciting ∼1500 R*/rod/s (Fig. 5*A*). The magnitude response functions (Fig. 5*B*) to 3- and 6-Hz flickers exhibited a non-monotonic relationship with illumination intensity. However, the responses to 12 Hz grew monotonically with illumination intensity. For Rho^P23H/+^ retinas, the crossover at 6 Hz is at ∼800 R*/rod/s and in agreement with the values determined for the *in vivo* ERG (Fig. 4*A*). The magnitudes of the Rho^+/−^ and control responses to 6-Hz flicker diverged from the control values at low intensities and did not crossover (Fig. 5*C*), following the same trend that was observed under *in vivo* conditions (Fig. 4*B*). These results indicate that changes in the photoreceptors other than collecting area determine how the TCS magnitude functions respond to the genetic manipulations of Rho^P23H/+^ and Rho^+/−^ mice.

### Analysis of the mechanisms underlying the increase in the magnitude of the flicker responses in Rho^P23H/+^ and Rho^+/−^ photoreceptors

**Figure 6.**
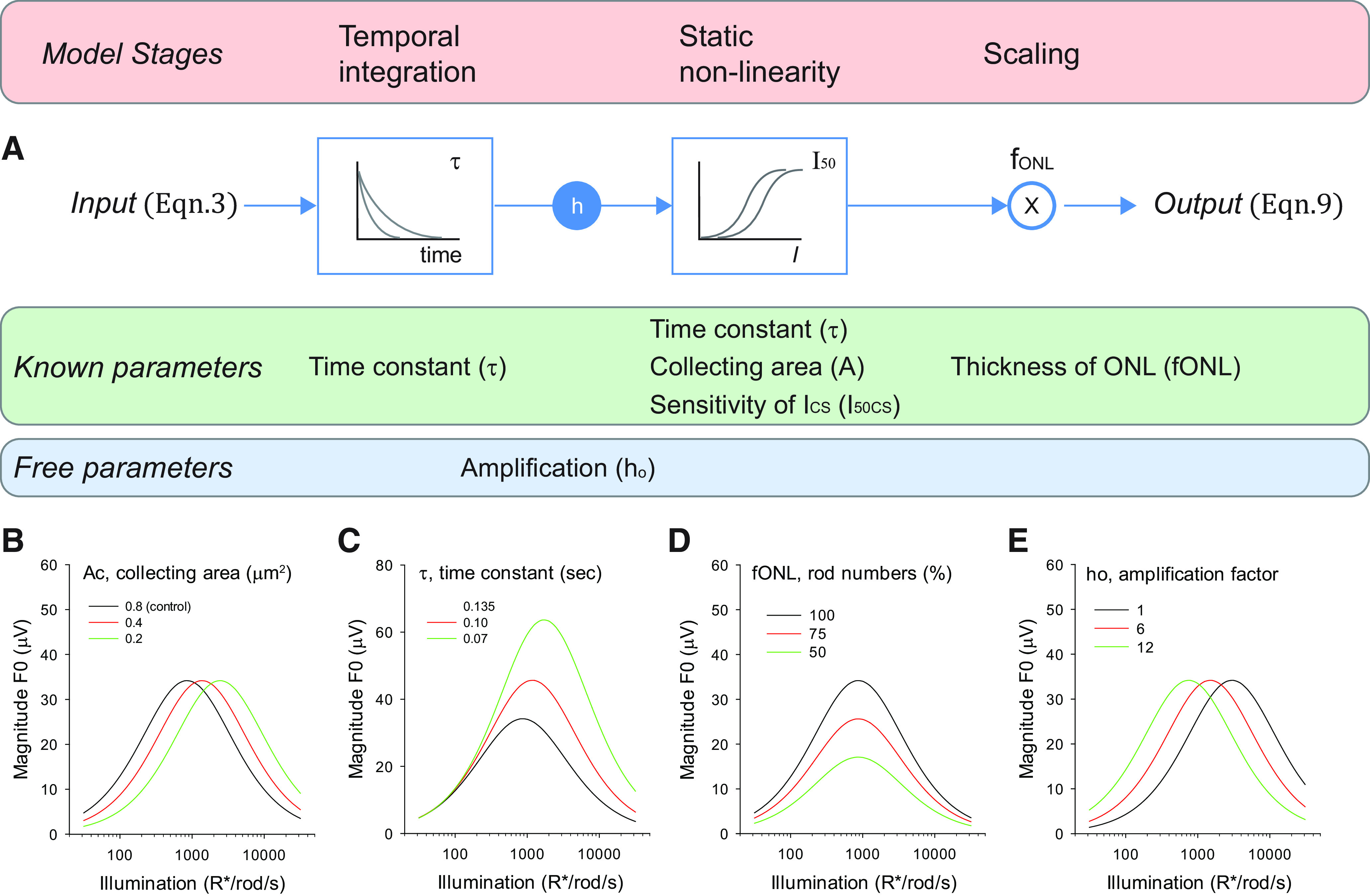
Potential mechanisms underlying the increase in the magnitude of the flicker responses in Rho^P23H/+^ and Rho^+/−^ photoreceptors. ***A***, The two-stage model of flicker responses consists of a first order exponential filter followed by a static non-linearity. The bandwidth of the filter depends on the value of the time constant (τ) while the position of the non-linearity along the intensity axis depends on the filter time constant (τ), the amplification factor (ho), the collecting area (A_c_) and the sensitivity of the circulating currents (I_50CS_). The output is scaled by the fractional thickness of the ONL (f_ONL_). Approximate values of τ, Ac, I_50CS_, and f_ONL_ were determined experimentally or obtained from the literature. The amplification factor (h_0_) is the only free parameter in the model. ***B–E***, Predicted model responses to 6-Hz flicker, 75% contrast using Equation 13 and the following control values for the model parameters: A_c_ = 0.87 μm^2^, τ = 0.136 s, f_ONL_ = 1, h_0_ = 4, EC_50_ = 300 ph/μm^2^/s. ***B***, The rod collecting area (A_c_) controls the position of the magnitude function along the intensity axis. ***C***, The photoresponse kinetics (τ) control the magnitude of the flicker responses in high but not in low background intensities (see text for details). ***D***, Number of photoreceptors, as represented by the relative thickness of the ONL (f_ONL_) scales the magnitude of the responses. ***E***, The multiplicative factor *ho*, which represents the sensitivity of the rod photoresponse, controls the activation range without introducing changes in the peak magnitude of the responses to contrast. Illumination levels are expressed in terms of the expected photoisomerization rate (R*/rod/s) for WT rods.

The magnitude response functions of Rho^+/−^ and Rho^P23H/+^ mice may be shaped by other factors: (1) changes in the photoresponse kinetics (Sakami et al., 2014); (2) changes in the number of rods because of degeneration and thinning of the ONL (Fig. 1; Sakami et al., 2011, 2014); or (3) changes in sensitivity (Sakami et al., 2014). To understand the relative contribution of each of these factors to the flicker responses, we applied a linear/nonlinear model that captures the major features of the isolated ERG responses driven by rod photoreceptors (Umino et al., 2019). This model has two stages (Fig. 6*A*; Eqs. 4, 9): a linear filter (associated with the temporal response of the rods) followed by a static non-linearity (associated with the suppression of the circulating current in rods).

Each of the candidate TCS-modulating factors controls a different aspect of the flicker magnitude function. The rod collecting area (A_c_) controls the position of the magnitude function along the intensity axis (Fig. 6*B*). As collecting area values decrease, the probability of absorbing incident photons also decreases and the functions shift to the right. The photoresponse kinetics (τ) control the magnitude of the flicker responses at high, but not low, background intensities (Fig. 6*C*). This is because faster kinetics (smaller τ) extend the bandwidth of the filter (increasing the relative magnitude of the responses to high frequencies; Eq. 5; for details see Umino et al., 2019) and also reduces the integration time of the photon response (Eq. 6). The remaining two factors in the model play straightforward roles. The number of photoreceptors, as inferred from the thickness of the ONL (f_ONL_), scales the magnitude of the responses (Fig. 6*D*), while the multiplicative factor ho, which represents the sensitivity of the rod photoresponse, controls the activation range but without changing the peak magnitude of the responses (Fig. 6*E*).

The parameter values are readily determined with this simple model. f_ONL_ is measured directly from Figure 1, and A_c_ is estimated from published reports (Tables 1, 2). The filter time constant follows from the τ_D_ of rods (Umino et al., 2019). As a measure of the relative position of the non-linearity, we use I_50CS_, the sensitivity of the suppression of the circulating currents by steady background lights (Umino et al., 2019). The only free variable in the model is the amplification factor (ho). Next, we measured and quantified the photoresponse sensitivity and kinetics of Rho^+/−^ and Rho^P23H/+^ retinas to determine the values of the remaining parameters in the model.

### Faster rod photoresponse recovery kinetics in Rho^P23H/+^ and Rho^+/−^ mice

**Figure 7.**
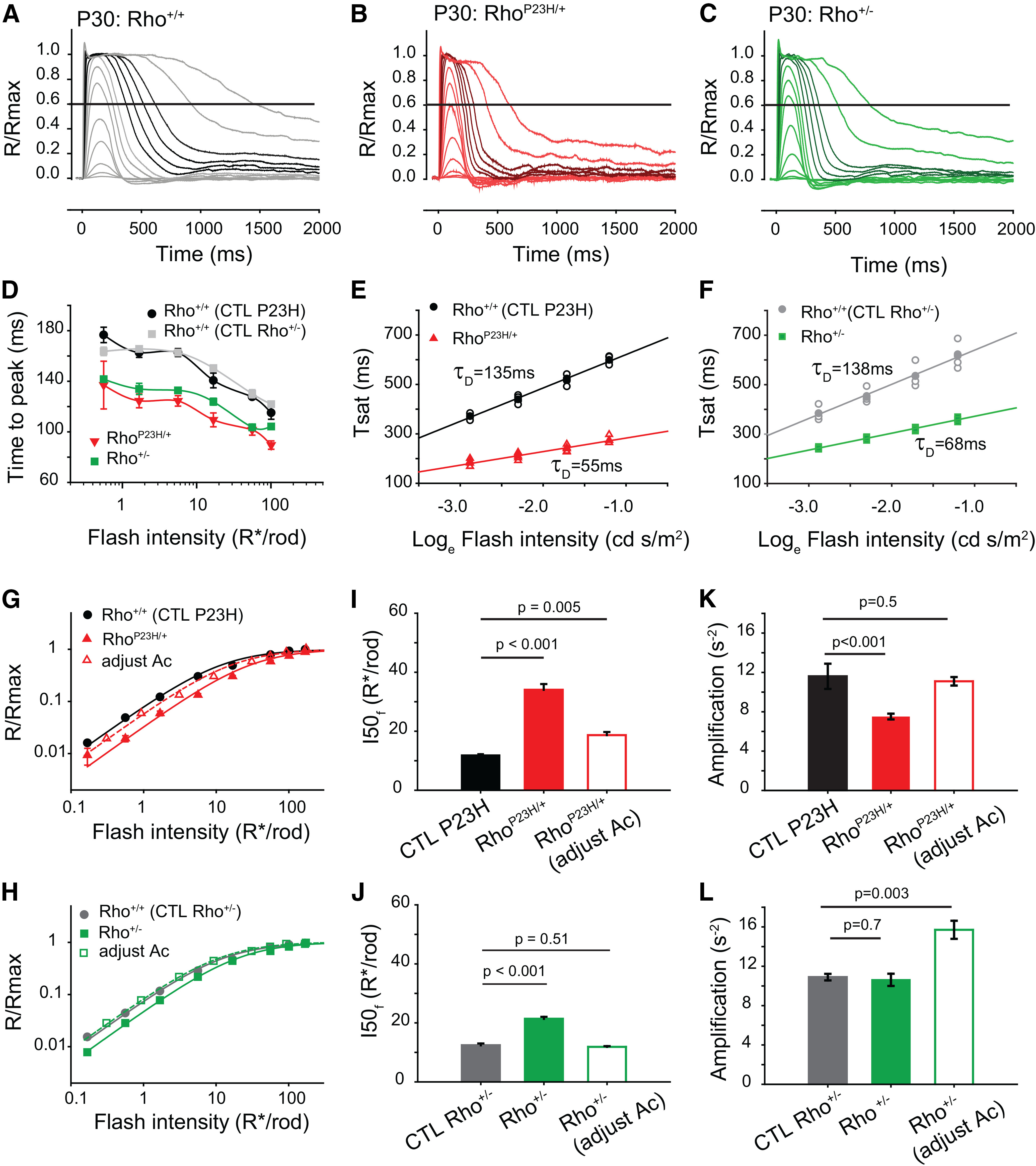
Rho^P23H/+^ and Rho^+/−^ mice exhibit faster rod recovery kinetics than control mice. ***A–C***, Representative dark-adapted flash responses of isolated photoreceptors measured by *ex vivo* transretinal ERG. Responses are from (***A***) P30 Rho^+/+^, (***B***) Rho^P23H/+^, and (***C***) Rho^+/−^ retinas and normalized by the “steady response” during saturating flashes. Solid line indicates 60% recovery of the photoresponse used to estimate time in saturation (T_sat_) in ***E, F***. The four darker colored responses in each panel were used to calculate τ_D_ in ***E***, ***F***. ***D***, Average time-to-peak values for Rho^+/+^, Rho^P23H/+^, and Rho^+/−^ retinas plotted as function of photoisomerizations elicited per flash in control rods. Responses of control retinas speed up gradually with flash intensity. Times-to-peak of Rho^P23H/+^ and Rho^+/−^ retinas were 25–30 ms faster than those of controls. Symbols represent mean ± SEM. ***E***, ***F***, T_sat_ of photoresponse recovery as a function of the natural log of the flash intensity of Rho^+/+^ control retinas (CTL P23H and CTL Rho^+/−^, respectively) compared with (***E***) Rho^P23H/+^ retinas and (***F***) Rho^+/−^ retinas. Points in the linear range of saturation (see dark lines in corresponding panels above) were used to determine τ_D_ for each mouse line (Vinberg and Koskelainen, 2010). Filled symbols and bars represent mean ± SEM (error bars smaller than symbols), and open symbols represent measurements from individual retinas. *N* = 4 retinas from at least two mice. ***G***, ***H***, Flash intensity versus response functions for Rho^+/+^ sibling control (CTL P23H) and Rho^P23H/+^ retinas (***G***) and Rho^+/+^ controls (CTL Rho^+/−^) and Rho^+/−^ retinas (***H***). The data were well fit (*R*^2^ > 0.98) with Hille functions (Hille coefficient = 1), suggesting linearity of the responses with dim flashes. Open symbols indicate the responses of Rho^P23H/+^ retinas and Rho^+/−^ retinas after accounting for differences in collecting area indicated in Table 1. Flash intensities on the *x*-axis indicate photoisomerizations/rod in control retinas. ***I***, ***J***, Average I50f values for the Hille functions fitting the flash intensity of Rho^P23H/+^ retinas and their sibling controls (***I***) and Rho^+/−^ retinas and their sibling controls (***J***). Open bars represent I50f values after adjusting for differences in collecting areas listed in Table 1. Bars represent mean ± SEM (*n* = 4 for each genotype). Statistical analysis: double-tailed *t* tests, *p* values are indicated on plots. ***K***, ***L***, Average amplification values for Rho^P23H/+^ retinas and their sibling controls (***K***) and Rho^+/−^ retinas and their respective sibling controls (***L***). Open bars represent amplification values after adjusting for differences in collecting areas listed in Table 1. Bars represent mean ± SEM (*n* = 4 for each genotype). Statistical analysis: double-tailed *t* tests, *p* values indicated on plots. Intensity levels are expressed in terms of the expected photoisomerization rate (R*/rod) for WT rods.

We used the *ex vivo* transretinal ERG preparation to isolate and measure rod photoresponses (Sakami et al., 2014; Vinberg et al., 2014; Vinberg and Kefalov, 2015; see Materials and Methods) and characterize the photoresponse kinetics. The responses of P30 Rho^P23H/+^ and Rho^+/−^ retinas to brief flashes recovered to baseline more quickly than those of their age-matched sibling controls (Fig. 7*A–C*). Times-to-peak were 30–40 ms faster both in Rho^P23H/+^ and Rho^+/−^ retinas compared with their respective controls (Fig. 7*D*). To quantify the recovery kinetics, we determined the τ_D_ of recovery of the *ex vivo* ERG by measuring the time for the saturating responses (Fig. 7*A–C*, dark traces) to recover to ∼60% of their “steady” plateau maximum (Vinberg and Koskelainen, 2010). We plotted the saturation time as a function of the natural logarithm of the flash strength (Fig. 7*E*,*F*); the slope of this relationship reflects the τ_D_ of the *ex vivo* ERG (Vinberg and Koskelainen, 2010). The τ_D_ values of both groups of control mice were similar (135 ± 4.5 and 138 ± 8.4 ms for Rho^+/+^ sibling controls of Rho^P23H/+^ and Rho^+/−^ mice, respectively) and comparable to τ_D_ values measured by *ex vivo* ERG recordings (∼166 ms; Vinberg and Koskelainen, 2010) as well as by *in vivo* paired-flash ERG recordings (∼125 ms; Peinado Allina et al., 2017). In contrast, the τ_D_ of Rho^P23H/+^ and Rho^+/−^ retinas were 55 ± 2.0 and 68 ± 2.2 ms, respectively, ∼2.0–2.5 times faster than that of their respective control retinas (*p* < 0.001, one-way ANOVA). These values are slightly faster than the dim flash recovery of Rho^P23H/+^ retinas measured at P14–P16 (Sakami et al., 2014) and in line with the kinetics measured by suction electrode recordings in Rho^+/−^ rods (Lem et al., 1999; Calvert et al., 2001).

The normalized intensity versus responses functions of P30 Rho^P23H/+^ and Rho^+/−^ retinas were shifted to the right and well fit with Hill functions (Fig. 7*G*,*H*). The half saturation value (I*_50f_*) of the flash responses was 2.8-fold higher in Rho^P23H/+^ retinas relative to control values (Fig. 7*I*) but was only 1.6-fold higher after adjusting for the smaller collecting areas of Rho^P23H/+^ rods (Table 1). These values are in line with previously reported losses in photosensitivity (per photon absorbed) in Rho^P23H/+^ rods (Sakami et al., 2014). Rho^+/−^ retinas exhibited normal half saturation values after adjusting for differences in collecting area (Fig. 7*J*), suggesting normal photosensitivity (per photon absorbed) in Rho^+/−^ retinas.

To determine whether the onset of the responses plays a role in the dissimilar photosensitivities of Rho^P23H/+^ and Rho^+/−^ retinas, we measured the amplification factor (Pugh and Lamb, 1993) in their transretinal ERG recordings (see Materials and Methods). The early phase of the responses (t < 75 ms, normalized amplitude < 0.2) fit with the amplification factor theory, with an *R*^2^ > 0.9 in all cases. Amplifications of control Rho^P23H/+^ and Rho^+/−^ littermates were 12.3 ± 0.25 and 10.9 ± 0.22 s^−2^, respectively, and comparable to values obtained by others using this technique (Vinberg et al., 2014). The amplification of Rho^P23H/+^ retinas was 11.1 ± 0.4 s^−2^ after adjusting for differences in collecting area (see Table 1) and not significantly different from control (two-tailed *t* test, *p* = 0.5, *n* = 4; Fig. 7*K*). Interestingly, after adjusting for collecting area differences, the amplification factor of Rho^+/−^ mice was 15.7 ± 0.9 s^−2^, ∼1.5-fold larger than that of control mice (two-tailed *t* test, *p* = 0.003; Fig. 7*L*) and in line with the increase in amplification determined with suction electrode recordings (Calvert et al., 2001).

### Loss of sensitivity in the suppression of circulating currents by steady lights depends on collecting area and kinetics

**Figure 8.**
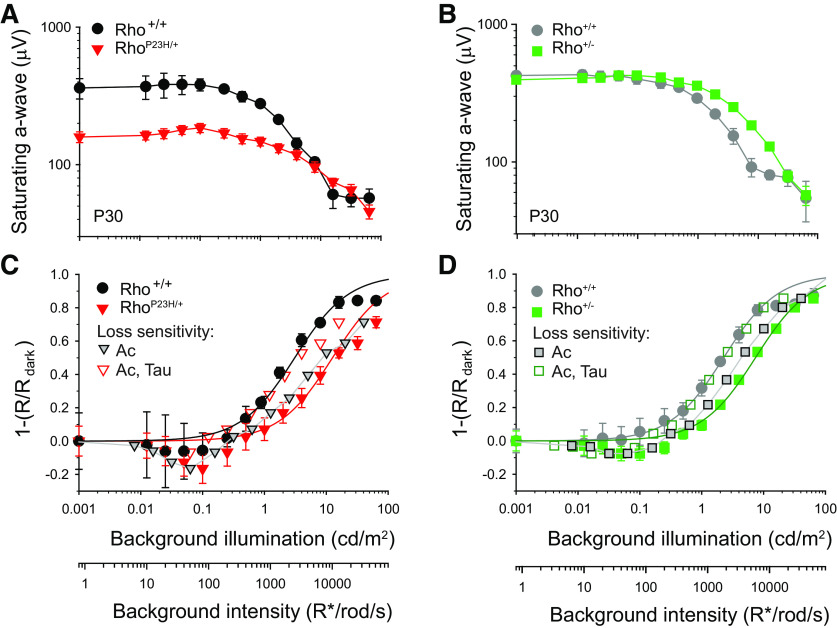
Desensitized suppression of circulating currents by steady lights. ***A***, ***B***, Maximum a-wave amplitude in response to a saturating flash stimulus of 1776 cd*s/m^2^ as a function of background intensity for (***A***) P30 Rho^P23H/+^ and their sibling Rho^+/+^ control mice and (***B***) P30 Rho^+/−^ and their sibling control Rho^+/+^ mice. ***C***, ***D***, Corresponding steady state circulating current (I_circ_) suppression data at each background intensity determined by subtracting normalized saturating flash responses from 1. Solid fit lines represent Hill equation fits of the measured responses. Open symbols account for the loss in sensitivity arising from faster kinetics (τ) and reduced collecting area (A_c_) in Rho^P23H/+^ (***C***) and Rho^+/−^ (***D***) mice after applying Equation 12 and values listed in Table 2. The loss in sensitivity attributed to differences in kinetics shifted the data to the right by 2.5-fold in Rho^P23H/+^ retinas (depicted by the transition from the curve labeled with open to the curve with gray triangles) and 2.0-fold in Rho^+/−^ retinas (transition from curve labeled with open squares to curve with gray squares). The remaining loss in sensitivity is attributed to reduced collecting area and shifted the curves to the right by 1.6-fold (gray triangles to full triangles in Rho^P23H/+^ retinas; gray squares to green squares in Rho^+/−^ retinas). For all panels, symbols represent mean ± SEM (in some cases, error bars are smaller than symbols), *n* = 6 Rho^P23H/+^ mice and *n* = 4 Rho^+/+^ controls, *n* = 6 Rho^+/−^ mice and *n* = 6 Rho^+/+^ controls.

Rod photoresponse recovery kinetics control the sensitivity of the rod circulating currents (I_50CS_) to steady background illumination (Fortenbach et al., 2015). In turn, I_50CS_ controls the dynamic range of the flicker responses (Umino et al., 2019). Here, we used an *in vivo* saturating flash ERG protocol (see Materials and Methods; Lyubarsky et al., 1999) to determine how rod photoresponse recovery kinetics and collecting areas control I_50CS_ in Rho^P23H/+^ and Rho^+/−^ retinas. In this protocol, a bright flash (∼1800 cd*s/m^2^) that elicits a maximal ERG a-wave amplitude was superimposed on differing levels of steady background illumination. Under these conditions, the amplitude of the a-wave provides a measure of the amount of circulating current remaining at each background illumination level (Lyubarsky et al., 1999). Tests were performed with Rho^P23H/+^ and Rho^+/−^ mice (and their respective Rho^+/+^ controls) at P30 (Fig. 8*A*,*B*). At dim background intensities (<3 cd/m^2^), the responses of Rho^P23H/+^ mice were significantly reduced in amplitude compared with controls (Fig. 8*A*), most likely as a result of retinal degeneration (see Fig. 1). In contrast, the maximal a-wave responses of Rho^+/−^ mice were similar in amplitude to those of control mice at dim background intensities (Fig. 8*B*), consistent with minimal degeneration. Additionally, as background levels increased from darkness to ∼0.1 cd/m^2^, we observed a slight increment in the responses of Rho^P23H/+^ (<20%) and Rho^+/−^ (<10%) mice. Further increases in background levels resulted in a monotonic reduction in a-wave amplitudes. The departure of the responses from the asymptote at the brightest background illumination levels (32 and 64 cd/m^2^) may reflect the activation of cone responses at higher illumination levels (Lyubarsky et al., 1999). Thus, the responses at 32 and 64 cd/m^2^ were not included in the fits and analysis described below.

Steady state suppression of circulating currents in control retinas followed a simple, saturating trend that was well described by sigmoidal functions (see Materials and Methods) with I_50CS_ values of ∼2480 (Fig. 8*C*, black line) and ∼1800 R*/rod/s (Fig. 8*D*, dark gray line). Circulating current suppression functions of both Rho^P23H/+^ and Rho^+/−^ mice are shifted to the right relative to those of control mice. Fits with sigmoidal functions indicate I_50CS_ values of ∼10,100 (Fig. 8*C*, red lines) and ∼6000 R*/rod/s (Fig. 8*D*, green lines) in Rho^P23H/+^ and Rho^+/−^ retinas, respectively, consistent with 4.1-fold and 3.3-fold losses in I_50CS_ relative to control mice. The loss in sensitivity can be accounted by the reduced collecting areas and the τ_D_s (as a measure of rod recovery kinetics) in Rho^P23H/+^ and Rho^+/−^ mice (Eqs. 13, 14; Table 2; Fig. 8*C*,*D*, open symbols).

### A quantitative model predicts an increase in the magnitude of flicker responses in Rho^P23H/+^ and Rho^+/−^ rods

We applied maximum likelihood estimation (Millar, 2011) to determine whether the linear nonlinear model (Eq. 9; Fig. 6) can explain the enhanced photoresponses of Rho^P23H/+^ and Rho^+/−^ retinas recorded with the transretinal ERG (see Fig. 5). The fits to 3-, 6-, and 12-Hz flicker responses were evaluated independently and the corresponding values of *h_0_*, the single free variable in the model, are listed in Table 3. Model parameters and their values are listed in Table 2.

**Figure 9.**
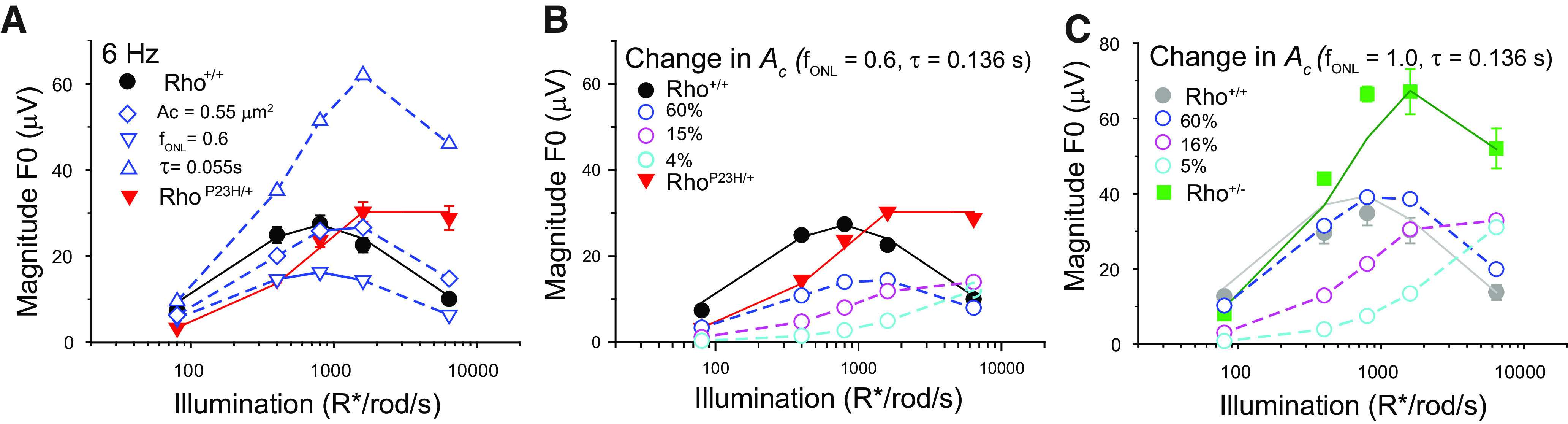
Predicted magnitude responses with the model of Rho^+/+^ retinas to 6-Hz flicker, 75% in contrast, assuming (***A***) single changes in a single parameter. The parameters under study were the collecting area (A_c_), the thickness of the ONL (f_ONL_), or the response kinetics (τ). Each curve (open symbols) represents the magnitude function estimated when the indicated parameter was assigned the corresponding value measured in Rho^P23H/+^ retinas while the remaining parameters had control values (see Table 2). For reference, the full symbols represent responses of Rho^+/+^ and Rho^P23H/+^ retinas (from Fig. 5). ***B***, Systematic changes in A_c_ for retinas with 60% degeneration (as per Rho^P23H/+^ retinas; Fig. 1) and normal response kinetics (τ = 0.136 s; Fig. 7). ***C***, Systematic changes in A_c_ in retinas without degeneration (f_ONL_ = 1, as per Rho^+/−^ retinas; Fig. 3) and normal response kinetics (τ = 0.136 s; Fig. 7). Full symbols indicate magnitude response of Rho^+/+^ and Rho^+/−^ retinas (from Fig. 5). Only after accounting for fast photoresponse kinetics can the magnitudes responses of Rho^+/+^ retinas approximate those of Rho^P23H/+^ and Rho^+/−^ (see text). Illumination levels are expressed in terms of the expected photoisomerization rate (R*/rod/s) for WT rods.

We used this model to evaluate how collecting area, photoreceptor loss or response kinetics in Rho^P23H/+^ mice influence the responses of Rho^+/+^ mice to 6 Hz (Fig. 9*A*). Filled symbols and solid lines represent responses of P30 Rho^+/+^ and Rho^P23H/+^ retinas and model fits (from Fig. 5), whereas open symbols and dashed lines represent noted modelled effects. Assuming negligible degeneration (f_ONL_ = 1) and normal kinetics (τ = 0.136 ms), this model predicts that a reduction in collecting area (A_c_ decreased from 0.87 to 0.55 μm^2^; Table 2) would shift the magnitude responses x1.6-fold along the intensity axis (Fig. 9*A*, open blue diamonds). By contrast, degeneration of the outer retina (f_ONL_ decrease from 1 to 0.6; Table 2) would scale down the magnitude of the response (Fig. 9*A*, open blue inverse triangles), while faster kinetics (τ decrease from 0.136 to 0.055 ms; Table 2) without degeneration would selectively enhance the magnitude of the responses at illumination levels >100 R*/rod/s (Fig. 9*A*, blue triangles). The individual changes in any one of these factors cannot explain the measured responses of Rho^P23H/+^ retinas (Fig. 9*A*, red symbols).

It is possible that our estimation of the collecting area in Rho^P23H/+^ mice is too large, for example by disruption of disk stacking. Sufficiently small A_c_ values can shift the magnitudes to the right, as observed for the magnitude functions of Rho^P23H/+^ retinas. However, in Rho^P23H/+^ retinas the magnitude responses are scaled down by the degeneration factor (f_ONL_ = 0.6) and fall short of explaining the responses, independent of the collecting area value (Fig. 9*B*). In view of the critical role that degeneration plays in the analysis, we repeated the analysis in Rho^+/−^ retinas, which have minimal retinal degeneration (f_ONL_ = 1.0). In this case, reductions in *A_c_* shift the functions to the right, while preserving their magnitudes. This behavior does not match the response magnitudes observed in Rho^+/−^ retinas, which are significantly higher than control retinas (Fig. 9*C*). The increase in contrast sensitivity predicted by manipulating τ parameters in the model (Figs. 6*C*, 9*A*) does match the magnitude responses (Fig. 5) and highlights the significance that rod recovery kinetics play in the enhancement of flicker response magnitude in Rho^P23H/+^ and Rho^+/−^ retinas.

## Discussion

In this study, we demonstrate that the fast rod photoresponse recovery kinetics of Rho^P23H/+^ mice result in enhanced retinal and behavioral TCS at mesopic light levels between the ages of P30 and P90, despite significant photoreceptor degeneration and reduced flash ERG responses. The magnitude of the increased retinal TCS depended on flicker frequency, with larger responses at high (6–12 Hz) compared with those at low (1.5–3 Hz) flicker frequencies, and was mediated by rods and not cones (Fig. 2). In mouse, retinal and behavioral responses to flicker at mesopic light levels, as in this study, are driven largely by rods (Umino et al., 2019; Pasquale et al., 2020), while cones drive visual sensitivity to light increments (Naarendorp et al., 2010). Enhanced retinal and optomotor TCS also preceded retinal degeneration in Rho^+/−^ mice, a slower model of degeneration with fast photoresponse recovery kinetics. Therefore, our findings have important implications for our understanding of temporal vision during retinal degenerative processes.

### TCS of Rho^P23H/+^ mice increases as ONL thickness and flash ERG responses decrease

As retinal degeneration advanced, Rho^P23H/+^ mice exhibited a progressive enhancement in retinal and behavioral TCS compared with controls (Fig. 2). The increase in retinal TCS was greatest at high frequency flicker (6–12 Hz) and progressed with age, until the ONL thickness decreased by 70% compared with controls. The increase in TCS is in stark contrast to the sharp decline in flash ERG a- and b-wave amplitudes over the same time period (Fig. 1). A recent study describes a similar relative retention of optomotor TCS of Rho^P23H/+^ mice at dim light levels and low temporal frequencies, hypothesized to be a result of adaptive homeostatic plasticity mechanisms allowing the maintenance of scotopic vision (Leinonen et al., 2020). However, our results suggest that the increase in TCS of Rho^P23H/+^ mice under mesopic conditions is driven primarily by changes in rod photoresponse properties.

### Enhanced TCS is associated with fast rod photoresponse recovery kinetics

Our observation that Rho^+/−^ mice (Lem et al., 1999), a mouse model that has little retinal degeneration and little if any loss of their flash ERG responses, exhibited a significant increase in retinal TCS rules out degeneration or remodeling as a required mechanism underlying the increase in retinal TCS. Furthermore, the increase in the magnitude of the rod photoreceptor responses to flickering stimuli of both Rho^P23H/+^ and Rho^+/−^ mice isolated with the *ex vivo* transretinal ERG confirm that retinal remodelling is not required for enhanced TCS responses.

We previously demonstrated that fast rod recovery kinetics underlie enhanced TCS in R9AP overexpressing mice (R9AP95; Umino et al., 2019). Here, we show that the increase in TCS in Rho^P23H/+^ and Rho^+/−^ mice is largely a result of the faster rod photoresponse recovery kinetics in these mice. We demonstrate that both Rho^P23H/+^ and Rho^+/−^ mice share important response properties with R9AP95 mice, consistent with the notion that faster response kinetics underlie their increase in TCS: (1) faster response kinetics; (2) a loss in the sensitivity of the steady state circulating current suppression (I_50CS_); (3) an associated shift in the peak flicker responses of the full and isolated ERG; and (4) larger rod responses to temporal contrast.

Once rod loss, collecting area, and response kinetics are accounted for (Fig. 9), our model with both linear and nonlinear components (Umino et al., 2019) successfully and quantitatively explains the increase in TCS in Rho^P23H/+^ and Rho^+/−^ retinas (Fig. 5). Rod loss scales the magnitude of the response, collecting area and kinetics control the position of the magnitude functions along the intensity axis, while response kinetics determines the magntidue of the response to high intensities (Fig. 6).

Our model operates under the assumption that Rho^P23H/+^ and control retinas share the same sensitivity values (equal h_0_ values for each genotype). Flash sensitivities (in the dark) differ by a factor of 1.6 (Fig. 7*G*,*I*), and including this factor into the model did not significantly improve the fits (data not shown). This suggests that relatively small differences in flash sensitivity do not influence the magnitude of the flicker responses. The differences in h_0_ values across frequencies (Table 3) may not represent frequency-dependent differences in sensitivity; rather, they more likely reflect the limited attenuation of the filter used to model the temporal responses of the rod photoreceptor. Our simple model provides valuable insights into the role of collecting area, photoreceptor kinetics and photoreceptor loss in the flicker response of rods, and a more detailed model of the rod photoresponses with three or more integrating stages (Robson and Frishman, 2014) is needed to meet the strong attenuation of rod responses at high temporal frequencies.

Dim responses are shaped by the amplification kinetics of the forward transduction cascade and the kinetics of inactivation (Nikonov et al., 2000). Normal amplification in Rho^P23H/+^ retinas (Fig. 7*K*) suggests that the (1.6-fold) reduction in flash photosensitivity (Fig. 7*I*; Sakami et al., 2014) can be attributed to their faster rod inactivation kinetics (Fig. 7*E*). The high amplification in Rho^+/−^ rods (Fig. 7*L*) may counteract their fast inactivation kinetics (Fig. 7*F*) to produce normal photosensitivity values (Fig. 7*J*). The faster photoresponses of P30 Rho^+/−^ rods are caused by a reduction in rhodopsin density and faster collision rates of phototransduction proteins on the outer segment disk membrane (Lem et al., 1999; Calvert et al., 2001; Makino et al., 2012). However, the density of rhodopsin in older (P90) Rho^+/−^ mice is thought to be normal (Liang et al., 2004), suggesting that the faster recovery kinetics of rods in older Rho^+/−^ mice are a result of an alternative mechanism(s) related to changes in the volume of the rod outer segment, accelerated exchanges of cations, or altered expression of inactivation proteins relative to outer segment volume (Liang et al., 2004).

The mechanism(s) underlying the faster photoresponses of rods in P30 Rho^P23H/+^ mice (Sakami et al., 2014; Fig. 7) are also unclear. Photoreceptors of P30 Rho^P23H/+^ mice have reduced rhodopsin expression and reduced outer segment volume (Sakami et al., 2011, 2014), which could contribute to the faster photoresponse recovery kinetics via similar mechanisms as described for P90 Rho^+/−^ rods (see above). Another possibility is that the fast recovery kinetics in Rho^P23H/+^ mice could be a result of a change in Ca^2+^ dynamics, which act to accelerate photoresponse recovery in light-adapted conditions (Pugh et al., 1999; Fain et al., 2001). In addition, the upregulation of ER stress responses observed in degenerating P23H rods (Sung et al., 1991; Kaushal and Khorana, 1994; Illing et al., 2002; Saliba et al., 2002; Lin et al., 2007; Gorbatyuk et al., 2010; Adekeye et al., 2014; Chiang et al., 2015) could trigger Ca^2+^ release from the ER (Sizova et al., 2014), resulting in increased intracellular Ca^2+^ concentration. However, this increase would likely cause a slowing of the response, because light-induced acceleration is caused by a reduction in intracellular Ca^2+^ concentration (Pugh et al., 1999). Further studies are required to unveil the mechanisms that underlie the fast response kinetics in Rho^P23H/+^ rods.

### Frequency-dependent loss in TCS of Rho^P23H/+^ mice

The enhanced TCS of Rho^P23H/+^ and Rho^+/−^ mice at mesopic light levels is largely a result of accelerated rod photoresponse kinetics (Fig. 2). However, it is not clear what drives the frequency-dependent loss in TCS of Rho^P23H/+^ mice. It may be that degenerating rods first lose their innate ability to respond to low temporal frequencies before high temporal frequencies. In this regard, it was recently demonstrated using behavior (Pasquale et al., 2020) that the illumination level at which rod-driven, TCS saturates is not absolute: rod-driven TCS saturates at dimmer light levels at low temporal frequencies compared with rod driven TCS at high temporal frequencies. The brighter light levels required to elicit enhanced flicker ERG responses of Rho^P23H/+^ mice at advanced stages of degeneration (Fig. 2*H*) may be approaching the saturation range for rod driven TCS to low temporal frequencies, resulting in a smaller response to low frequencies than higher frequencies. In addition, the frequency-dependent loss in TCS may result from disruption of one or more of the parallel retinal circuits that are active at the mesopic intensities where we observe enhanced TCS in Rho^P23H/+^ mice (for review, see Sharpe and Stockman, 1999; Bloomfield and Dacheux, 2001; Grimes et al., 2018).

In conclusion, the rod dysfunction of fast photoresponse kinetics is present in many animal models of retinal degeneration [Rho^G90D/+^ mice (Sieving et al., 2001); Rho^P347LorS/+^ pigs (Kraft et al., 2005); Rho^+/−^ mice (Lem et al., 1999; Calvert et al., 2001; Liang et al., 2004); and RPE65^−/−^ (Woodruff et al., 2003)]. Moreover, accelerated rod recovery kinetics are present in some human adRP patients (Wen et al., 2011). Diagnosis of advanced rod dysfunction in humans has been performed using full-field ERG, full-field stimulus test, and full-field perimetry (Bennett et al., 2017). Measurement of retinal or behavioral TCS could prove useful as a rapid quantitative test indicative of early signs of rod dysfunction. An important next step is to determine whether enhanced TCS, at mesopic light levels, can be observed in human patients with RP. Such tests may require focal measurements in the peripheral retina, where rods outnumber cones, combined with colorimetric methods that independently control the response of rods and cones in mesopic light conditions (Zele and Cao, 2014).

## Synthesis

Reviewing Editor: Arianna Maffei, SUNY Stony Brook Decisions are customarily a result of the Reviewing Editor and the peer reviewers coming together and discussing their recommendations until a consensus is reached. When revisions are invited, a fact-based synthesis statement explaining their decision and outlining what is needed to prepare a revision will be listed below. The following reviewer(s) agreed to reveal their identity: NONE. Note: If this manuscript was transferred from JNeurosci and a decision was made to accept the manuscript without peer review, a brief statement to this effect will instead be what is listed below.

The authors thoroughly addressed the reviewers' critiques by adding new data, figures and analysis. The study provides an important contribution to the field, it is technically rigorous and well presented.
